# End-user research into understanding perceptions of and reactions to a microarray patch (MAP) for contraception among women in Ghana, Kenya and Uganda

**DOI:** 10.3389/frph.2024.1351692

**Published:** 2024-03-07

**Authors:** Moushira El-Sahn, Rose Elliott, Mona El-Sahn, Jeff Lucas, Trisha Wood Santos

**Affiliations:** ^1^ Routes2Results, Sevenoaks, United Kingdom; ^2^ Routes2Results, Aberdeen, United Kingdom; ^3^ Routes2Results, London, United Kingdom; ^4^ Trisha Wood Santos Consulting, LLC, Seattle, WA, United States

**Keywords:** family planning, Africa, social behavioural research, microneedles, acceptability, products in development, MAP developers, MAP attributes

## Abstract

**Introduction:**

Many organizations are developing new contraceptive products and approaches that promote self-care including a microarray patch (MAP) that has the potential for self-administration with appropriate training. We studied women's perceptions of the MAP technology with the primary goal of providing feedback on product attributes to inform early technical design decisions regarding various MAP contraceptive products in development by MAP developers.

**Methods:**

Our study consisted of a qualitative phase with in-person In-Depth Interviews (IDIs) with a total of 60 women of reproductive age (WRA) and quantitative surveys, via face-to-face computer-assisted interviews of a total of 927 women in Ghana, Kenya and Uganda. Women's perceptions on 12 attributes of the MAP were assessed through written descriptions, a profile, and visual stimuli such as graphics and images.

**Results:**

Overall, the most widely preferred attribute set included: a hand-applied MAP, utilizing one circular patch, with a sticky backing, no larger than 2 cm diameter in size, applied by self, to the arm, offering sensory feedback (clicking sound and/or color change signals) to confirm enough pressure, successful application and removal, lasting 6 months with up to 12 months return to natural state of fertility. There is space to allow for variation in MAP designs (including the use of an applicator or provider administered MAP) if the design promotes and reflects the needs and expectations of users and providers.

**Discussion:**

The contraceptive MAP had a high and broad level of appeal amongst all groups of women who participated in the study and has a strong value proposition around important contraceptive needs such as ease of use, convenience, and discretion.

## 1 Introduction

The Sustainable Development Goals call for universal access to sexual and reproductive health care services including increasing the proportion of women of reproductive age (WRA) (15–49 years) who have their family planning needs satisfied by use of a modern contraceptive method (1). Despite progress to meet this goal, just over half (56%) of WRA who wanted to avoid pregnancy in sub-Saharan Africa were using a modern method in 2021 (2). The World Health Organization (WHO) recommends self-care, or “the ability of individuals, families and communities to promote their own health, prevent disease, maintain health, and to cope with illness and disability with or without the support of a health worker” as an innovative way to increase access to primary health care while minimizing strain on the health care facilities and workers (3). Self-care has the potential to increase access to modern contraception and support women's empowerment by allowing discreet access and use in preferred locations like a pharmacy or private home, as well as to start and stop using contraception when they want to do so (4).

Many organizations are developing new contraceptive products and approaches that promote self-care including microarray patches (MAPs) that have the potential for self-administration with appropriate training. The MAP technology comprises microneedles, or micro-sized tips, attached to a backing material and applied to the skin using a finger or applicator allowing for transdermal drug delivery (5) (Supplementary Figure S1). The MAP technology is being studied for use in several indications including vaccines, HIV prevention drugs and long-acting contraception (5–15).

Research to understand end-user perspectives is important to incorporate early and often in the development of new products, but has not always been practiced in the sexual and reproductive health field (16–20). Product characteristics such as duration of action, form (e.g., pill, injection), presence and magnitude of side effects, user- vs. provider-administration and many other factors like risk perception, social and cultural norms and costs influence whether someone will use and continue a method (16, 17, 20, 21). Over the past decade, there has been an effort to study and incorporate end-user preferences into the design of new contraceptives, HIV prevention products and multi-purpose prevention technologies (MPTs) (16–18, 20–25). The results of these studies indicate several areas of overlap including preferences for discretion, longer duration, and ease of use and are relevant when considering use of a MAP platform for contraception, HIV prevention or a combination of both (24–31).

Our study's primary objective was to provide feedback to MAP developers on MAPs' product-based attributes to inform early technical design. The secondary objective was to understand what respondents thought about MAPs and what they saw as its value from the various stimuli, the Consumer Target Product Profile (CTPP), and product attributes respondents were shown. The third objective was to understand interest and intent to try, as well as whether respondents thought MAPs was a better contraceptive option than those they knew of. Given the proprietary nature of product development, this publication does not include specific information on any one product but provides overall suggestions for researchers and practitioners to consider when developing a contraceptive MAP.

The study was conducted in Ghana, Kenya, and Uganda and included qualitative and quantitative methodologies to triangulate the findings and generate insights. These three countries were selected to represent voices from both East and West Africa with varying rates of contraceptive use and method mix, wherein the depot medroxyprogesterone acetate (DMPA) contraceptive injection is one of the most used methods (32).

## 2 Methods

### 2.1 Overview of project

**Figure 1:**
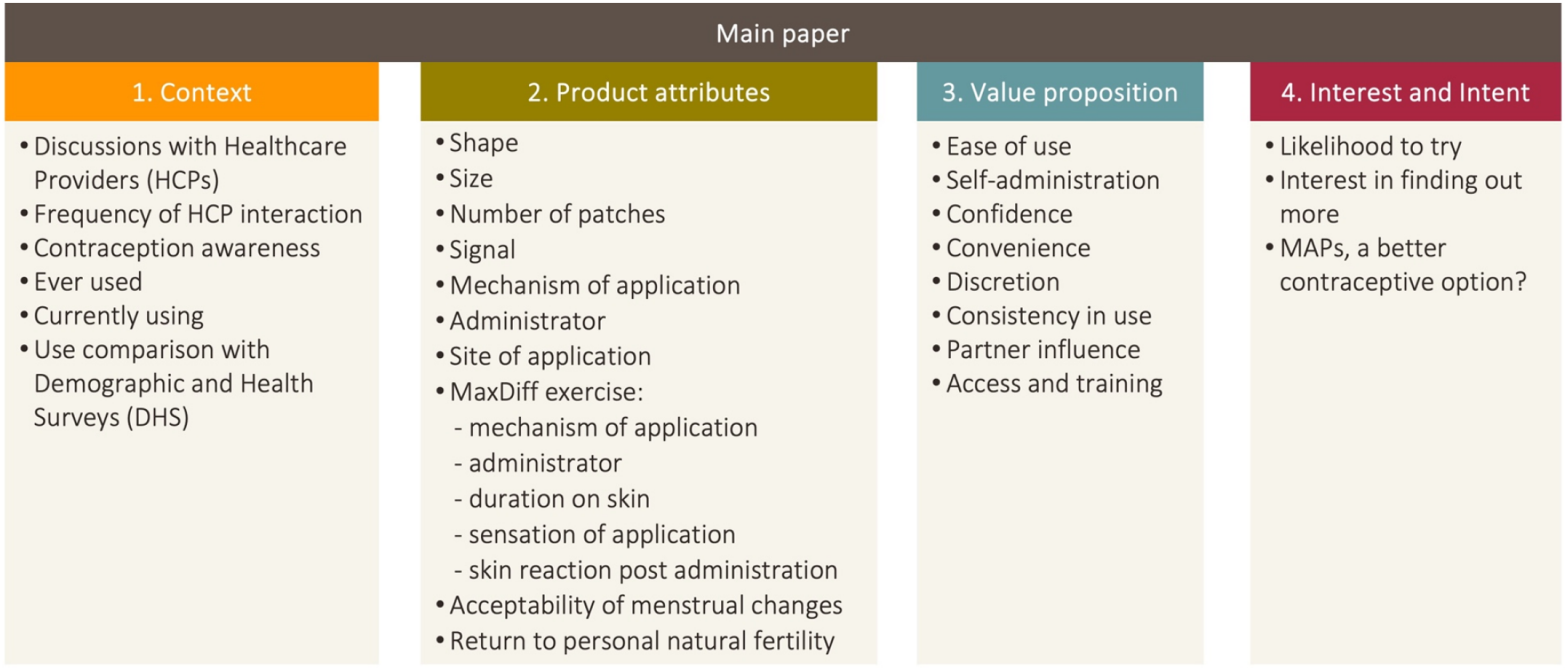
Research study results and findings flow.

Figure 1 shows the flow of the research study results and findings. The results of the research will follow the objectives laid out in the methodology of this paper, with the inclusion of an upfront section based on results which illustrate the environment and context of the respondents who took part in this study.

Our study consisted of two phases: a qualitative phase with in-person In-Depth Interviews (IDIs), followed by a quantitative phase upon the completion of the qualitative research and analysis, via face-to-face computer-assisted interviews (CAPI). Respondents that participated in the qualitative component did not participate in the quantitative component. All interviews were conducted at a place of the respondent's choosing, often in their home, or a space they felt they could talk openly. The primary objective sought to gauge women's perceptions on 12 attributes of the MAP were assessed through written descriptions, a profile, and visual stimuli such as graphics and images. The secondary objective of this research naturally overlaps with the first objective. A value proposition is how a product or product attributes can fill relevant end-user needs. Therefore, the value of a product naturally builds on and from the product attributes themselves.

### 2.2 Qualitative phase

#### 2.2.1 Sample and data collection

Data were collected via in-person IDIs with a total of 60 women in Ghana, Kenya and Uganda. The target population consisted of WRA, who self-reported as being sexually active (with a man in the last 3 months), not currently pregnant and not planning to conceive in the next year, open to using contraceptives/family planning, and in Socio Economic Class (SEC) C-D. This research utilized the EquityTool which is a short, country-specific questionnaire to measure relative wealth (34). SEC strata C and D were selected for this research as they encompass the broadest and largest section of the population, and are critical target populations for contraceptive programs. Eighteen to 40 was selected as the age range for the qualitative sample as an upper limit of 40 was considered to be able to provide an adequate representation of the older age range among childbearing women in qualitative work. Aside from the eligibility criteria detailed above, there were no quotas to subdivide the sample, and characteristics of the sample depended on the natural fallout following recruitment. For the qualitative component, discussions lasted between 40 and 60 minutes with the consent of respondents in face-to-face one-on-one IDIs. For data collection, interviews were audio-recorded, transcribed, and, where necessary, translated prior to analysis.

#### 2.2.2 Data analysis

For the qualitative data, a coding framework was developed by the study team following a primary round of reviews of the first set of transcripts. This initial framework was then used to code all remaining transcripts fully, with scope to broaden and clarify the initial code frame as more transcripts were reviewed and analyzed. The third round of analysis identified key themes that emerged from the data, as well as points of difference. An iterative and systematic process of content and pattern analysis was carried out. The study team used the analytical categories developed as part of the coding framework to derive meaning from the various pieces of evidence to answer the research questions.

### 2.3 Quantitative phase

#### 2.3.1 Sample and data collection

The quantitative phase took place subsequently to the qualitative phase. Data were collected via face-to-face CAPI of a total of 927 women in Ghana, Kenya and Uganda. The target population for the quantitative phase mirrored that of the qualitative phase; the age range, however, of 18–49 was chosen, to reflect recommendations by the WHO for survey research among WRA (35). Other demographic information was captured during the interviews (Table 1); however, this did not determine eligibility.

**Table 1:** Demographic and motherhood-related data, self-reported at quantitative phase of this study (%).

Demographics				
	Total (*n* = 927)	Ghana (*n* = 315)	Kenya (*n* = 303)	Uganda (*n* = 309)
Age (%)				
18–23 years old	23.9	27.9	23.1	20.7
24–30 years old	40.1	37.5	39.3	43.7
31–35 years old	18.2	17.8	18.8	18.1
36–48 years old	17.7	16.8	18.8	17.5
Mean (years)	28.95	28.46	29.32	29.06
Education level (%)				
Primary + below	16.1	14.0	18.8	15.5
Junior secondary	17.8	27.3^b^	7.9	17.8
Senior secondary	41.4	43.5	51.2	29.8^a^
Higher education	23.8	14	23	36
Religion (%)				
Practicing	87.1	91.4	90.1	79.6^a^
Non-practicing	12.5	8.3	9.2	20.1^b^
Christian	34.0	47.0	46.5	8.4^a^
Protestant	21.1	4.1^a^	24.1	35.6
Catholic	16.5	5.4	12.2	32.0^b^
Pentecostal	16.2	31.7^b^	8.3	8.1
Muslim	11.5	10.8	8.3	15.5
Other	0.3	0.6	2	0.3
No religion	0.3	0.3	0.7	–
Relationship status (%)				
Married live*	53.6	37.1	70.3^b^	54.0
Single + BFs***	17.0	23.5^b^	13.9	13.6
Not living together	10.4	12.7	7.3	11.0
Living together	9.6	15.6^b^	3.3	9.7
Married not live**	4.6	3.5	2.0	8.4^b^
Single + BF(s)***	17.0	23.5^b^	13.9	13.6
Single no BF(s)***	3.6	7.0^b^	2.0	1.6
Widowed	0.9	0.6	1.0	1.0
Divorced	0.3	–	0.3	0.6
Motherhood				
	Total (*n* = 927)	Ghana (*n* = 315)	Kenya (*n* = 303)	Uganda (*n* = 309)
Number of pregnancies (%)				
None	18.3	27.6^b^	13.9	13.3
1	24.8	21.3	27.4	25.9
2	24.5	21.3	24.8	27.5
3	17.8	16.5	20.1	16.8
4	8.3	6.3	6.6	12.0^b^
5	4.4	5.1	5.0	3.2
6	1.8	1.9	2.3	1.3
Mean	1.95	1.75^a^	2.05	2.06
Number of children (%)				
None	20.9	32.1^b^	15.2	15.2
1	26.3	21.0^a^	28.7	29.4
2	25.4	20.3^a^	28.4	27.5
3	16.3	15.2	17.2	16.5
4+	11.1	11.4	10.6	11.3
Mean	1.76	1.60	1.85	1.83
Pregnancy desire (%)				
Yes, next 2 years.	28.4	27.9	22.8	34.3
Yes, >2 years.	53.1	59.4	47.9	51.8
I don’t want any	18.6	12.7	29.4	13.9

^a^Significantly lower than the 2 other countries.

^b^Significantly higher than the 2 other countries.

* Married living with partner.

** Married not living with partner.

*** Boy Friend(s).

For the quantitative phase, women were surveyed via face-to-face computer-assisted 30-minute interviews. For data collection, mobile phones/tablets with offline data storage capability were used and data were automatically uploaded when an internet connection was available.

#### 2.3.2 MaxDiff

For the quantitative research study, a MaxDiff exercise was co-created through consultations with MAP product developers, product development experts, and contraception experts and researchers. A pre-defined set of product attributes were selected, and within each attribute a number of variables (on how it would perform or act) were developed. These attributes were considered important to inform product development in aspects where variation was possible and where end-users' perceptions were unknown. The exercise was approximately halfway through the interview after respondents had read and thought about the CTPP.

The MaxDiff methodology is a useful tool to extract least and most motivating attributes. Respondents were shown a series of six screens, where each screen presented one set of two options and respondents indicated which was more and which was less motivating. Scoring was then calculated based on the number of times an option was shown *and* selected as less motivating and more motivating.

#### 2.3.3 Data analysis

The closed-ended quantitative data were analyzed initially by total base size including an examination of data at a total respondent level and at a country level. Following this, quantitative data were analyzed by key groups such as marital status, across age groups, and urban vs. rural.

In accordance with the conventional acceptance of statistical significance at a *P*-value of 0.05 or 5%, confidence intervals (CI) are frequently calculated at a confidence level of 95%. Tests used in analysis included paired/overlap *T*-Test for means and paired/overlap *Z*-Test for percentages. In general, if an observed result is statistically significant at a *P*-value of 0.05, then the null hypothesis should not fall within the 95% CI. Statistical significance (*P* < 0.05) is observed when data in one country is different from findings in the other that are not due to chance. There were, however, no significant differences between sample groups (demographics such as age, setting, contraceptive use etc.,) which were relevant to the objectives. Results from the qualitative research are given to provide context to the quantitative results.

Perceived ease of administration, interest, intent and whether respondents felt the MAP was an improvement on current contraception they were aware of, will be presented including Top 2 Box scores (T2B or “positive” scoring) and Bottom 2 Box (B2B or “negative” scoring) scores as a way of looking at the Likert scale questions. The Top 2 Box score is a method utilized within the market research industry of summarizing the positive responses (the two most positive options in a Likert scale question), and the Bottom 2 Box scores are a way of summarizing the negative responses (the two most negative options in a Likert scale question). This paper does not wholly rely on Top 2 Box or Bottom 2 Box scoring, and will also present each score for every rating statement within the overall scale.

Where predefined lists of reasons were provided in the quantitative questionnaire, these were written based on answers from the qualitative phase, with an “Other—specify” option also included.

Open-ended data within the quantitative interviews were also analyzed. The process began with review of verbatim responses for each question. Common themes were identified, as well as factors associated with each theme. This represented a code frame. Each verbatim response was then analyzed and assigned to its appropriate code.

### 2.4 Shared methodology

#### 2.4.1 Recruitment methods

For each country, specific urban and rural locations were selected with three key locations in each market (Table 2). These locations were selected to provide cultural and social variances and geographical diversity. Within each of these locations, the research was conducted in the main urban area and also surrounding rural regions. A 50-50 split between urban and rural was achieved with the aim of providing an urban and rural view of each country.

**Table 2:** Sample across qualitative and quantitative phases of this study (*n* = 987).

	Total	Ghana	Kenya	Uganda
Overall sample				
Total	60	20	20	20
Region 1: Abokobi (Ghana), Ndumberi (Kenya), Kiyindi (Uganda)	30	10	10	10
Region 2: Juaben (Ghana), Muhoroni (Kenya), Masese (Uganda)	30	10	10	10
Quantitative sample across regions				
Total	927	315	303	309
Region 1: Accra (Ghana), Nairobi (Kenya), Kampala (Uganda)	303	100	100	103
Region 2: Akokobi (Ghana), Kisumu (Kenya), Jinja Uganda)	185	100	42	43
Region 3: Takoradi (Ghana), Muhoroni (Kenya), Mbarara (Uganda)	134	51	41	42
Region 4: Juabeb (Ghana), Mombasa (Kenya), Kyanja (Uganda)	112	31	40	41
Region 5: Kumasi (Ghana), Ndumberi (Kenya), Masese (Uganda)	107	27	40	40
Region 6: Ukunda (Uganda only)	40	–	40	–

The recruitment process was consistent across all three countries and all regions. The target population was recruited using screening questionnaires (programmed and conducted on CAPI devices), which set out eligibility to take part in the research. All recruitment teams were female-led, and respondents were recruited from low-income areas. Specific households were selected through a random walk process (from a landmark such as a school, hospital or similar) whereby the teams attempt door-to-door screening with skips between houses (three dwellings in a rural area and five in an urban area).

#### 2.4.2 Stimuli

Participants in both parts of the study were shown the same stimuli: an overview of the MAP based on five showcards and moderator script (Supplementary Figure S1); an end-user-friendly profile (CTPP) read alongside the moderator/interviewer who would check in on understanding throughout (Supplementary Figure S2); images depicting potential patch appearance: shape (Supplementary Figure S3), size (Supplementary Figure S4), application site (Supplementary Figure S5), applicator types (due to intellectual property rights, applicator type images cannot be reproduced in this article) and a show-card presenting options of duration of pregnancy prevention against time to potential return to personal natural fertility (RTF) (Supplementary Figure S6). All stimuli were translated, and translations were offered throughout the interview to ensure optimal understanding of the research questions, and maximize ease of conversation for the respondent. Stimuli were always shown to all participants in the same order.

#### 2.4.3 Translations

All informed consent forms, stimuli and research tools were translated into the main languages spoken in the areas where fieldwork was conducted (Twi in Ghana, Kiswahili in Kenya, Luganda in Uganda). Respondents were able to choose languages for written materials and discussion, and to switch if preferred.

#### 2.4.4 Data collection

All interviews were conducted by interviewers from Ask Afrika and their in-country offices who were trained in market research methodologies and were native speakers. The research teams (comprising female interviewers and recruiters and mixed-sex supervisors) were briefed and trained, and pilot interviews were observed across all countries ensuring adherence to objectives, processes and ethical considerations.

#### 2.4.5 COVID-19

Due to the COVID-19 pandemic, precautions were put in place to minimize risk of viral transmission. Inter-country travel was minimized; the study team briefed the in-country fieldwork teams remotely using video meeting technology. Fieldwork was conducted only when there were no government restrictions in place which would be contravened by carrying out this work. Guidelines were developed based on those provided for face-to-face interviewing by the European Society for Opinion and Marketing Research (ESOMAR) (35). Provision was made for the use of alternative formats for completing the research such as telephone or video conferencing tools, if needed, to comply with government restrictions; in the event, however, it was possible to conduct all research face-to-face.

## 3 Results

### 3.1 Context

Table 1 illustrates the demographic make-up, including age, education level, religion, relationship status, number of pregnancies, children, and desire for more children in the next two years from the quantitative survey. Table 2 illustrates the achieved sample for qualitative and quantitative phases of this study across the regions per country.

### 3.2 Interaction with healthcare practitioner (HCP) about/accessing contraception

#### 3.2.1 Quantitative

Table 3 respondents were asked whether they have ever talked to an HCP about contraceptives. HCPs were described to respondents as including a doctor, a nurse, a nurse-midwife or community healthcare worker in a clinic or elsewhere. In Ghana more than half (59.4%, significantly high) of respondents reported not having any discussions with HCPs, compared to just under a quarter in Kenya and Uganda (24.1% and 23.6% respectively). Of those that reported having discussions with HCPs, these mostly happened in a clinic setting [Ghana 31.7%; Kenya 70.0% (significantly high); Uganda 48.2%]. Respondents in Uganda reported having a much higher frequency of interaction with HCPs (7.5 times on average in the last 12 months, significantly high), compared to 2.1 times (Ghana) and 1.5 times (Kenya).

**Table 3:** Interaction with HCPs—conversations about contraception, frequency and where most recent contraceptive was obtained (%).

	Total	Ghana	Kenya	Uganda
Total sample (*n*=)	927	315	303	309
Interaction with HCPs—conversations about contraceptives (%)				
Yes, in a clinic	49.7	31.7	70.0^b^	48.2
No, never spoken to HCP	35.9	59.4^b^	24.1	23.6
Yes, elsewhere, not in a clinic	14.3	8.9	5.9	28.2^b^
Frequency of conversations with HCPs in past 12 months—about contraceptives (#) mean/median number of discussions in the past 12 months				
Sample (*n*=)	461	100	212	149
In a clinic (mean)	2.36	2.1	1.5	7.5^b^
Sample (*n*=)	133	28	18	87
Elsewhere, not in a clinic (median)	2.0	2.0	1.0	2.0
Where most recent contraceptive was obtained (%) of women that named a recent contraceptive				
Sample (*n*=)	478	102	196	180
Healthcare facility	62.3	41.2	64.8	73.9
Pharmacy/drug store	26.6	36.3	27.0	20.6
Do not know/no answer	6.3	12.7	6.1	2.5
In the community	4.0	9.8	2.0	2.8
Setting: public or private for location of most recently obtained contraceptive (%)				
Public	71.9	78.6	78.0	75.4
Private	28.1	21.4	22.0	36.1

^a^ Significantly lower than the 2 other countries.

^b^ Significantly higher than the 2 other countries.

In Kenya and Uganda, most obtained their current contraceptives in a healthcare facility (66% and 74% respectively), and there was a split in Ghana between healthcare facility and pharmacy (41% vs. 36% respectively). The majority of healthcare facilities accessed were in a public setting across all three countries, at 71.9% overall (Ghana 78.6%, Kenya 78.0%, Uganda 63.9%) whereas 28.1% overall accessed contraceptives from a private facility (28.1% in Ghana, 21.4% in Kenya and 36.1% Uganda).

### 3.3 Contraceptive awareness

Male condoms had the highest level of awareness across all countries (Table 4) (83.2% in Ghana, 96.0% in Kenya and 75.4% in Uganda). This was followed by the oral contraceptive pill (70.5% in Ghana, 94.7% in Kenya and 85.8% in Uganda). The third most selected method was the contraceptive injection (68.6% Ghana, 96.0% in Kenya and 87.1% in Uganda). Kenya generally had significantly higher awareness levels than Ghana and Uganda across nearly all (10 out of 15) methods, except the Contraceptive Vaginal Ring (CVR) DMPA and DMPA sub cutaneous (DMPA-SC) injectables. Conversely, Ghana had significantly lower percentages of respondents reporting awareness of listed methods (7 out of 15).

**Table 4:** Aided awareness of contraception/methods to prevent pregnancy/family planning (%).

	Total	Ghana	Kenya	Uganda
Aided awareness of contraceptive methods (%)				
Sample (*n*=)	927	315	303	309
Male condom	84.8	83.2	96.0^b^	75.4
Oral daily contraceptive pill	83.5	70.5^a^	94.7^b^	85.8
Contraceptive injection	83.7	68.6^a^	96.0^b^	87.1
Oral emergency contraceptive pill	65.9	62.9	83.5^b^	51.8^a^
Contraceptive implant/rod	69.9	48.9^a^	88.4^b^	73.1
Female condom	58.0	50.2	80.5^b^	44.0
Intrauterine device (IUD)	48.8	25.1^a^	65.3^b^	56.6
Counting days	30.9	26.7	42.2^b^	23.9
Hormonal IUD	28.5	15.6^a^	38.9^b^	31.4
Contraceptive vaginal ring (CVR)	25.6	14.9^a^	26.7	35.3^b^
Withdrawal	23.7	22.9	27.1	21.4
Contraceptive patch	14.9	12.7	19.8^b^	12.3
Depot medroxyprogesterone acetate sub cutaneous (DMPA-SC)	13.5	5.7^a^	13.9	21.0^b^
Diaphragm/cap	11.4	11.1	13.2	10.0
Spermicide	10.0	7.3	9.2	13.6
Other methods	1.6	0.3	1.3	3.2
None — I have never heard of any methods	0.1	0.3	–	–

^a^Significantly lower than the 2 other countries.

^b^Significantly higher than the 2 other countries.

### 3.4 Contraceptive use

A third (30.7% in Kenya and 30.0% Uganda) to just under half (47.3% in Ghana (significantly high) were not currently using a contraceptive (Table 5). Overall, 12.3% stated they had discontinued their contraceptive use. Ghana had a significantly higher proportion of respondents (20.3%) who had discontinued their contraceptive compared to Kenya and Uganda (5.3% and 11.0% respectively). Male condom was the most ever used method by 60.5% of respondents overall (52.9% in Ghana, 64.7% in Kenya and 64.1% in Uganda). The next most ever used contraceptive was the contraceptive injection which was selected by 43.1% of respondents overall [26.8% in Ghana (significantly low), 55.8% in Kenya and 47.2% in Uganda]. Over a third of the overall sample reported ever use of the oral daily pill and oral emergency pill (35.4% and 33.3% respectively).

**Table 5:** Ever and current use of contraception.

	Total	Ghana		Kenya		Uganda	
Total Sample (*n*=)	927	315		303		309	
Ever used contraceptive (%) [methods used by over 2% of sample listed]							
Male condom	60.5	52.9		64.7		64.1	
Contraceptive injection	43.1	26.8^a^		55.8		47.2	
Oral daily contraceptive pill	35.4	27.1^a^		40.3		39.2	
Oral emergency contraceptive pill	33.3	26.8^a^		55.8		47.2	
Contraceptive implant	25.1	15.0^a^		34.7^b^		25.9	
Counting days	23.7	24.8		27.4		18.8	
Withdrawal	17.8	19.4		19.5		14.6	
IUD	6.8	2.5^a^		8.3		9.7	
Female condom	5.1	4.8		7.3		3.2	
None	3.7	7.6^b^		1.3		1.9	
DMPA-SC	2.6	1.0		1.3		5.5^b^	
Hormonal IUD	2.4	1.6		3.3		2.3	
CVR	2.3	1.0		1.0		4.9^b^	
Currently used contraceptive (%) [methods used by over 2% of sample listed]							
Demographic Data (DHS)*	–	DHS 2014		DHS 2020		DHS 2016	
Modern contraceptive use by all women^†^, DHS %							
		18.2		42.0		27.3	
Total R2R**		DHS	R2R	DHS	R2R	DHS	R2R
Contraceptive injection	18.0	6.0	12.7^a^	13.6	20.8	13.9	20.7
Contraceptive implant	12.6	3.7	6.7^a^	13.2	18.5	4.7	12.9
Male condom	9.9	2.0	11.4	3.8	9.6	3.1	8.7
Oral daily contraceptive pill	9.6	3.9	6.7	5.3	10.6	1.5	11.7
Oral emergency contraceptive pill	5.4	^‡^	10.3^b^	0.6	3.0	0.1	2.9
Counting days	4.4	3.1	6.7	3.7	4.6	1.2	1.9
Withdrawal	3.6	1.4	4.1	1.1	3.6	1.9	2.9
I am not currently using	36.1	77.2	47.3^b^	53.4	30.0	69.7	30.7

^a^Significantly lower than the 2 other countries.

^b^Significantly higher than the 2 other countries.

* DHS data refers to USAID’s Demographic and Health Surveys Program data.

** R2R stands for Routes2Results, data from this study.

^†^ DHS data relates to women from 15 to 49 years, whereas R2R data collected included women from the age of 18 to 49 years, and participants in the R2R study were screened into the research on the basis that they were sexually active, and open to using contraceptives.

^‡^ No data available.

Current use for any specific contraceptive method was low. The injection was the most currently used method by 18.0% of respondents overall [12.7% in Ghana (significantly low) and 20.8% in Kenya and 20.7% in Uganda], with the vast majority (72.7% overall) using DMPA Intermuscular (DMPA-IM) three-monthly injection. Between a third (Kenya and Uganda) or nearly half (Ghana) of the respondents in this study were not currently using anything to prevent an unwanted pregnancy. However, Demographic and Health Survey (DHS) data from all countries shows higher percentages of women not currently using from 53.5% (Kenya) to 77.2% (Ghana) (36–38).

### 3.5 Primary objective

#### 3.5.1 MAP attributes

Women's perceptions on 12 attributes of the MAP were assessed. These were: shape of the patch, size of the patch, the number of patches, sensory feedback mechanisms/signals to confirm correct usage, mechanism of application, preferred administrator, site of application, duration on skin, sensation of application, administration site reactions, menstrual changes and return to personal natural fertility level.

#### 3.5.2 Shape

In both phases, respondents were shown two possible MAP shapes: a circle and a rectangle (Supplementary Figure S3).

##### 3.5.2.1 Qualitative

In the qualitative component of the study, views on shape were mixed. Respondents were asked about whether they had any preferences on shape, and around half indicated that they did; even among those who specified a preferred shape, there was a moderately common view that it was not of great importance. Reasons for shape preference were similar across the actual shape options preferred: a perception that certain shapes (particularly the circle) were smaller, and easier to use. Some respondents mentioned that the shape was not important as compared to the functionality of the MAP.

##### 3.5.2.2 Quantitative

The quantitative results indicated that respondents had a strong preference for a circular patch (74.2% overall). This preference was present in all countries, but was more prevalent in Ghana and Kenya [85.7% (significantly high) and 73.9% of respondents respectively] than in Uganda (62.8% of respondents). Overall, 18.2% of respondents selected rectangle, which was driven by Uganda and Kenya (25.9% and 17.8% respectively), whereas a small percentage of respondents from Ghana selected rectangle (11.1%). Respondents were also given the answer option of “no preference”, which was selected by 7.65% overall [3.2% in Ghana (significantly low), 8.3% in Kenya and 11.3% in Uganda].

#### 3.5.3 Size

Respondents were shown in both the qualitative and quantitative phases three visual graphics representing the three possible sizes for a square MAP: 2 × 2 cm, 3 × 3 cm and 5 × 5 cm (Supplementary Figure S4).

##### 3.5.3.1 Qualitative

Respondents in all three countries expressed a preference for the smallest patch size; the main reasons given for this were discretion, both in terms of perceived discreet application (for example, potential irritation left post application would cover a smaller area) and fear of worse side effects with a larger patch size. It is of note, however, that almost all respondents indicated, when asked, that if the biggest patch were to be the only available option, they would still be willing to try the MAP on the grounds that it would fulfill the same function.

##### 3.5.3.2 Quantitative

In general, a smaller size was preferable. Three quarters of respondents in all countries (75.0% overall) preferred the smallest given option of 2 × 2 cm (76.8% in Ghana, 75.6% in Kenya and 72.5% in Uganda); the next smallest size, 3 × 3 cm, was preferred by around a quarter (26.3% overall) of respondents (25.1% in Ghana, 26.1% in Kenya and 27.8% in Uganda). The largest size, 5 × 5 cm, was preferred by a small minority (5.5% overall) of respondents [3.5% in Ghana, 2.0% in Kenya and 11.0% in Uganda (significantly high)].

#### 3.5.4 Number of patches and duration of protection

Across the quantitative and qualitative results, respondents reacted negatively to the idea of using multiple patches to achieve the same duration of protection (6 months) which had been proposed as being achieved by one patch, and to the idea of multiple patches in general.

##### 3.5.4.1 Qualitative

The majority of respondents indicated that multiple patches (2, 3, or 4) would not be acceptable, and the already limited acceptability declined as the number of patches increased, regardless of patch size. As with the idea of larger patches, respondents were concerned about the potential of increased side effects and possible discomfort at application, alongside the corresponding potential impact on discretion. The idea of multiple patches was felt to undermine the product's appeal of 6 months' continuous protection, its perceived simplicity with regard to ease of use, and the ability to maintain discretion around its usage.

##### 3.5.4.2 Quantitative

Respondents were asked whether three duration sets of pregnancy prevention (1 month, 3 months and 6 months) and three patch number options (one, two or three patches) were acceptable. Acceptability was discussed for each option set provided (Table 6). The most acceptable set, selected by 89.4% overall, was one patch to achieve 6 months of pregnancy prevention [87.6% in Ghana, 93.7% in Kenya (Significantly high), 87.1% in Uganda]. There was openness to one patch to achieve 3 months of pregnancy prevention which was the second most acceptable set, selected by 40.9% overall, (44.1% in Ghana, 42.6% in Kenya and 35.9% in Uganda). The remaining duration sets did not garner much acceptability support, however one patch to achieve 1 month of pregnancy prevention was selected as acceptable by a quarter (25.1%) overall, which was driven by Uganda (30.4% significantly high).

**Table 6:** Acceptability of number of patches (%).

	Total	Ghana	Kenya	Uganda
Total sample (*n*=)	927	315	303	309
1 patch to achieve 6 months of pregnancy prevention	89.4	87.6	93.7^b^	87.1
1 patch to achieve 3 months of pregnancy prevention	40.9	44.1	42.6	35.9
1 patch to achieve 1 month of pregnancy prevention	25.1	22.9	22.1	30.4^b^
2 patches to achieve 6 months of pregnancy prevention	22.2	11.4	37.3^b^	18.4
3 patches to achieve 6 months	13.3	5.4^a^	18.5	16.2

^a^ Significantly lower than the 2 other countries.

^b^ Significantly higher than the 2 other countries.

#### 3.5.5 Sensory feedback mechanisms

Respondents were asked to choose whether they would want sensory feedback mechanisms or signals at any of the following three stages: to indicate that the patch had been successfully applied to the body, whether enough pressure had been applied, and whether the patch could be removed.

##### 3.5.5.1 Qualitative

The clicking sound or color change was most preferred by respondents, depending on whether there is greater preference for associating correct application with sight (color change) or sound (click). The clicking sound was strongly associated with having applied enough pressure. It was perceived as useful in terms of alerting the user if they were to become distracted during the process of application, but there was a caveat from a minority of users around it not being acceptable if it were loud enough that it could potentially be overheard, risking the application being noticed by others. The color change was perceived to be discreet because of its lack of audibility. Concerns around the color change related to the potential of missing the signal if it were to appear and disappear rapidly. An image appearing on patch was viewed as being similar to color change and was less preferred as it did not serve any additional purpose.

##### 3.5.5.2 Quantitative

The signal choice set comprised color change, clicking sound, an image appearing on the patch or applicator and temporary dye appearing on the skin. Respondents were also able to choose all signals or no signal for any point; these options, however, were selected by a small minority of respondents (Table 7).

**Table 7:** Signal preference per application stage (%).

	Total	Ghana	Kenya	Uganda
Total sample (*n*=)	927	315	303	309
Successfully applied to the body				
Color change	43.4	45.1	42.6	42.1
Clicking sound	34.3	35.2	34.3	33.3
Images appears on skin	6.7	8.9	4.3	6.8
A dye appears on skin	4.3	4.8	5.9	2.3
All signals	5.4	2.9	7.6	5.8
No signal	4.3	1.0^a^	4.3	7.8
No preference	1.7	2.2	1.0	1.9
Enough pressure applied				
Color change	31.4	29.2	37.6^b^	27.5
Clicking sound	40.0	47.3	39.6	33.0^a^
Images appears on skin	12.0	11.7	7.9	16.2
A dye appears on skin	5.8	3.8	5.3	8.4
All signals	4.1	2.2	5.0	5.2
No signal	4.2	3.2	2.6	6.8
No preference	2.5	2.5	2.0	2.0
All steps complete—OK to remove				
Color change	33.5	40.0	36.0	24.6
Clicking sound	30.6	33.0	29.0	29.8
Images appears on skin	8.7	10.2	7.6	8.4
A dye appears on skin	5.6	3.6	10.2^b^	3.2
All signals	8.4	3.2	9.2	12.9
No signal	9.4	6.7	6.3	15.2^b^
No preference	3.7	3.5	1.7	5.8

^a^ Significantly lower than the 2 other countries.

^b^ Significantly higher than the 2 other countries.

As Table 7 demonstrates, it is important to provide some feedback mechanism at any stage, color change and a clicking sound were the two most preferred signals to indicate successful application of the MAP across all stages. There was a slight preference for color change at application (43.4% overall, which was significant different in Ghana and Uganda) and a slight preference for a clicking sound to indicate that enough pressure had been applied (40.0% overall, and significantly different in Ghana). There is a split preference overall towards color change and a clicking sound for the final stage, readiness for removal (33.5% and 30.6% overall, respectively).

#### 3.5.6 Mechanism of application

Respondents were presented with the choice of applying the MAP using an adhesive-backed patch which would be applied by hand and removed from the application site following the application process, or using an applicator (reusable or disposable) to apply the MAP.

##### 3.5.6.1 Qualitative

Reasons for wanting an adhesive-backed patch were related to its familiarity (in terms of its similarity to a sticking plaster), that it appeared less intimidating than an applicator, and that it was considered more discreet than an applicator. There was, however, a concern raised by some respondents regarding the adhesive-backed patch, relating to the challenge of applying the correct amount of pressure evenly across the surface of the patch.

The applicator was seen to address the concern around consistency of action, pressure and correct application. It was also considered to be faster than the patch, and by a minority of respondents, more hygienic. Concerns relating to the applicator were that it might be forceful and that it was seen to be potentially painful.

##### 3.5.6.2 Quantitative

(Table 8) most respondents (60.3%) preferred the adhesive-backed patch, with 34.1% preferring the applicator, and 5.6% having no preference. There were country differences, most respondents in Ghana and Kenya preferred the adhesive-backed patch [78.1% (significantly high) and 65.0% respectively; 37.5% in Uganda (significantly low)], with most respondents in Uganda preferring the applicator [52.1% (significantly high); 19.7% in Ghana (significantly low), 30.7% in Kenya].

**Table 8:** Preferred device type (%).

	Total	Ghana	Kenya	Uganda
Total sample (*n*=)	927	315	303	309
Adhesive (“sticky”) backed patch	60.3	78.1^b^	65.0	37.5^a^
Applicator	34.1	19.7^a^	30.7	52.1^b^
No preference	5.6	2.2	4.3	10.4^b^

^a^ Significantly lower than the 2 other countries.

^b^ Significantly higher than the 2 other countries.

### 3.6 Administration/administrator

#### 3.6.1 Most preferred

##### 3.6.1.1 Qualitative

Self-application was most preferred, followed closely by HCP application—particularly at the initial application, as HCPs were perceived as being able to educate and enable women to self-administer the MAP with confidence in the future. Respondents perceived self-application as discreet, because they could select when and where they chose to apply the MAP, without the involvement of anyone else. It was also perceived as convenient for the same reason; that no one else's assistance or involvement is required.

##### 3.6.1.2 Quantitative

A pre-defined list comprising self, partner, family member, friend and two sets of three HCP options (physician or nurse; community healthcare worker; pharmacist, either every time or HCP first or self thereafter) and an “Other—specify” option; HCP responses were grouped. Overall, 49.1% of respondents preferred self-administration (Table 9), which was driven by respondents in Ghana (81.1% significantly high) compared to respondents in Kenya 36.4% and Uganda 27.9% (significantly low). For respondents in Kenya, the same percentage, 36.0% (significantly high), selected self-administration after initial administration by a physician or a nurse, compared to Ghana (4.2% significantly low) and Uganda (10.3%). For respondents in Uganda, over a third preferred administration by a physician or a nurse (33.1% significantly high), whereas under a third preferred self-administration (27.9%).

**Table 9:** Preferred administrator of MAP (%).

	Total	Ghana	Kenya	Uganda
Total sample (*n*=)	927	315	303	309
I would administer it myself	49.1	81.1^b^	36.4	27.9^a^
A physician or nurse administers it the first time and then I would administer it to myself thereafter	16.4	4.2^a^	36.0^b^	10.3
A physician or nurse	16.0	2.5^a^	12.9	33.1^b^
My partner	5.6	8.1	3.0	5.5
A Community Healthcare Worker (CHW)	5.0	1.4	1.1	12.5^b^
A CHW administers it the first time and then I would administer it to myself thereafter	2.6	0.4^a^	2.7	4.8
A pharmacist	1.8	1.1	2.7	1.8
A pharmacist administers it the first time and then I would administer it to myself thereafter	1.5	–	3.8^b^	0.7
A friend	1.2	1.1	1.1	1.5
A family member	0.5	0.4	–	1.1
Other	0.4	–	0.4	0.7

^a^ Significantly lower than the 2 other countries.

^b^ Significantly higher than the 2 other countries.

#### 3.6.2 Least preferred

##### 3.6.2.1 Qualitative

Partners, family members and friends received the lowest preference. For partners, the reasons for this were that the partners may not have a high level of awareness of contraception and were not felt to be knowledgeable or reliable in terms of correct application, and that partners may not approve of using contraception. Family members and friends were felt not to be trustworthy in terms of confidentiality, in stark contrast to the view of HCPs; furthermore, respondents noted that family and friends may not support family planning, and as with partners, respondents were not confident that they would be able to apply the MAP better than they would themselves.

##### 3.6.2.2 Quantitative

Family was selected the least often (0.4% in Ghana, 0.0% Kenya, 0.7% in Uganda); friend was selected by 1.2% in all three countries; partner was selected by 5.6% overall (8.1% in Ghana, 3.0% in Kenya and 5.5% in Uganda).

#### 3.6.3 Site of application

Respondents were offered a pre-defined selection of potential application sites on the body and were asked to determine which sites were acceptable or unacceptable. Respondents were shown a women's body map designed specifically for this research (Supplementary Figure S5).

##### 3.6.3.1 Qualitative

**Figure 2:**
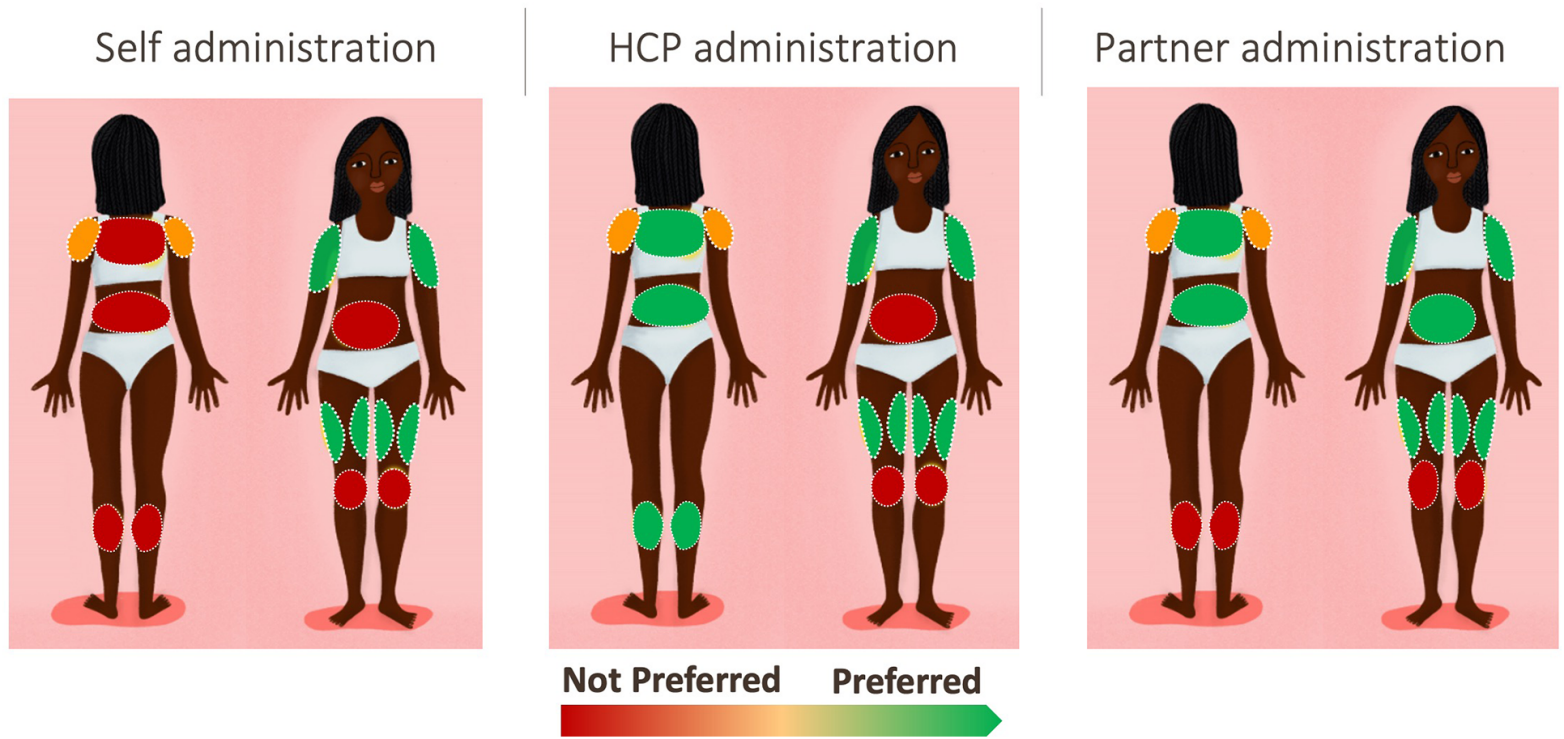
Preferred areas for administration, qualitative results.

Upper arms (with inner more preferred than outer, but both accepted) and thighs were the preferred application sites. Figure 2 illustrates the level of preference across the body map according to the person administering the MAP. Respondents noted that they felt confident that they could apply the MAP in these areas themselves with sufficient pressure, and that they were easy to reach, and the MAP could be applied sitting down to both areas. Furthermore, these areas are not frequently touched, not too sensitive and have the lowest perceived risk of post-application skin irritation. There was a split in opinion regarding the shoulder, in terms of whether it was perceived as an accessible area. The calf and back were perceived as hard to reach. A minority of respondents perceived that certain areas would create risk either to the MAP's efficacy or to the health of the respondent; the kneecap was seen as bony and uncomfortable, and there was concern that friction would impact the MAP, and the abdomen was viewed as delicate and close to important organs, leading to concern about how the medication would affect this area.

In terms of application by someone else, shoulder and knee were not preferred. As with self-administration, upper arms and thighs were preferred, with the addition of back and calf; for these areas, preference depended on privacy (in terms of coverage with clothing and how much clothing would need to be removed to allow access). For partner application, which was preferred by a minority of respondents, all areas were considered acceptable apart from knee and calf, with thigh and arms most preferred; the abdomen was acceptable only for partners, on the grounds that it is an area which the partner already sees.

##### 3.6.3.2 Quantitative

Respondents were asked to select which sites were acceptable for self-administration (Table 10); only self-administration was considered because the results from the qualitative phase indicated that self-administration was strongly preferred. Upper arms were the overall most preferred location. Upper outer arm was selected by 31.0% of respondents (24.8% in Ghana, 31.7% in Kenya and 36.6% in Uganda). Upper inner arm was selected by 29.1% of respondents (30.8% in Ghana, 30.4% in Kenya and 26.2% in Uganda).

**Table 10:** Preferred location for MAP application (%).

	Total	Ghana	Kenya	Uganda
Total sample (*n*=)	927	315	303	309
Upper arm (outside)	31.0	24.8	31.7	36.6
Upper arm (inside)	29.1	30.8	30.4	26.2
Upper inner thigh	10.7	12.4	10.2	9.4
Stomach (left or right)	9.3	10.5	11.6	5.8^a^
Upper outer thigh	9.1	5.4	8.3	13.6^b^
Shoulder	3.6	6.0	0.7	3.9
Knee	2.0	1.6	2.6	1.9
Upper back	1.9	2.5	1.7	1.6
Lower back (left or right)	1.8	4.4^b^	0.7	0.3
Calf	1.5	1.6	2.3	0.6

^a^ Significantly lower than the 2 other countries.

^b^ Significantly higher than the 2 other countries.

#### 3.6.4 MaxDiff exercise: mechanism of application, administrator, duration on skin, sensation of application and skin affect post administration

##### 3.6.4.1 Quantitative only

The results of the MaxDiff exercise are shown in Table 11. Among those tested, the most motivating attributes averaged across all countries were: a patch alone (47.8%), self-administered (56.0% overall), seconds on the skin (52.0%), causing a sensation of bristles or prickles against the skin, leaving small welts or scarring which disappears after a period of one day and with no visible skin irritation (65.7%). The least motivating attributes were overall: an applicator (44.2%), applied by a friend or family member (49.2%), staying on the skin for more than five minutes (59.7%), causing a pinching sensation (55.3%), visible grid of dots (52.4%) and redness, swelling that disappears after 3–4 days (51.9%).

**Table 11:** Most and least motivating attribute (%).

	Total		Ghana		Kenya		Uganda	
Total sample (*n*=)	927		315		303		309	
The MAP is administered with a(n) …								
	Most	Least	Most	Least	Most	Least	Most	Least
Patch (alone)	47.8	14.1	55.6	17.5	55.8	12.9	32.0^a^	12.0
Reusable applicator and patch	22.3	17.0	21.6	11.7	18.5	21.8	26.9	17.8
One-time applicator and patch	15.4	24.6	14.3	19.0	15.2	28.7	16.8	26.2
Applicator (no patch)	14.5	44.2	8.6	51.7	10.6	36.6	24.3^b^	44.0
Who administers the MAP								
Myself	56.0	12.9	74.3^b^	13.3	59.1^a^	7.3	34.3^a^	18.1
Trained provider (pharmacist and CHW*)	23.7	16.8	11.7^a^	21.6	27.4	16.2	32.4	12.6
My partner	10.4	21.0	6.0	18.7	6.6	20.8	18.4^b^	23.6
Chosen family/friend	9.9	49.2	7.9	46.3	6.9	55.8^b^	14.9^b^	45.6
How long the MAP stays on the skin …								
Matter of seconds	52.0	14.6	52.1	21.3	54.5	16.5	49.5	5.8^a^
<1 min	16.8	10.7	22.2^b^	7.6	14.9	7.3	13.3	17.2
1–5 min	17.8	15.1	13.0^a^	14.0	18.8	13.2	21.7	18.1
>5 min	13.4	59.7	13.0	57.1	11.9	63.0	15.5	58.9
Sensation of applying MAP is like …								
Prickles against your skin; briefly uncomfortable, but it can be ignored	69.1	14.0	76.8	17.1	75.9	12.9	54.7^a^	12.0
A group of pin pricks/scratches; cannot be ignored, may interfere with concentration	15.4	30.6	11.1	23.2	11.9	32.3	23.3^b^	36.6
A pinching sensation; cannot be ignored and may interfere with concentration	15.4	55.3	12.1	59.9^b^	12.2	54.8	22.0^b^	51.5
Post administration skin affect could be …								
There is no visible skin irritation after	65.7	11.2	70.8	15.9	69.3	7.9	57.0^a^	9.7
Small welts/scarring, disappears after 1 day	51.0	19.0	62.5^b^	21.0	50.2	22.1	40.1^a^	13.9
There may be a visible “grid of dots” after; no discomfort, disappear 1 h. −1 day	31.0	52.4	29.8	53.3	33.3	52.1	29.8	51.8
Small fluid droplets under the skin, but will disappear after 1 day	18.0	28.6	7.6	25.7	16.5	25.7	30.1^b^	34.3^b^
Redness, swelling, itching, discoloration, or pain immediately, disappears after 1 h	14.7	17.2	14.0	12.4	15.5	19.8	14.6	19.4
Redness, swelling … dis. after 3–4 days	9.9	51.9	7.0	50.5	5.0	57.4	17.8^b^	47.9
Redness, swelling … dis. after 1 day	9.7	19.7	8.3	21.3	10.2	14.9	10.7	23.0

^a^ Significantly lower than the 2 other countries.

^b^ Significantly higher than the 2 other countries.

In terms of differences between countries, the patch being selected as the most motivating option was driven by Ghana (55.6%) and Kenya (55.8%). In Uganda, while the patch was preferred (32.0%), relatively fewer respondents selected it as most motivating compared to the other two countries, and nearly a quarter (24.3%) selected most motivating for applicator variable. With regard to skin irritation and sensation, Ugandan respondents had significantly lower most motivating scores for bristles/prickles (54.7%) than Ghana (76.8%) and Kenya (75.9%). Self-administration is a clear motivating factor in Ghana (74.3%) and strong motivating factor in Kenya (59.1%). Although a motivator in Uganda (34.3%), respondents in Uganda also indicated that application by trained HCPs was also a motivating factor (32.4%).

Some product attributes were not included in the MaxDiff exercise and asked outside and after the exercise because, product attributes like side effects, menstrual changes, fertility, duration can overshadow other attributes. These attributes were either too early in development to understand variables, or the attribute/variable list were binary or unlikely to change.

#### 3.6.5 Acceptability of menstrual changes

##### 3.6.5.1 Quantitative

An inherent feature of giving a progestin continuously for 6 months is the possibility of changes to menstrual cycle and experience. Respondents were asked to assess the acceptability of pre-defined potential changes to their menstrual cycle from using the MAP, as follows: “It is possible that the microarray patch/MAP could cause changes to your monthly bleed/period. Would you find these changes acceptable or unacceptable, if they lasted beyond the first few months of use?”. The changes asked about were *No monthly bleed/period at all* and *Irregular periods*. Overall, having no bleed and irregular periods were unacceptable for many women (78.7% and 52.0% respectively; Table 12).

**Table 12:** Acceptability of menstrual changes (%).

	Total	Ghana	Kenya	Uganda
Total sample (*n*=)	927	315	303	309
No monthly bleed/period at all				
Unacceptable	78.7	91.4^b^	60.7^a^	83.5
Acceptable	21.3	8.6	39.3^b^	16.5
Irregular periods				
Unacceptable	52.0	45.4	51.2	59.5^b^
Acceptable	48.0	54.6	48.4	40.5^a^

^a^ Significantly lower than the 2 other countries.

^b^ Significantly higher than the 2 other countries.

#### 3.6.6 Duration of pregnancy prevention (PP) and return to fertility (RTF)

Respondents in both the qualitative and quantitative research phases were shown the same figure (Supplementary Figure S6) and were asked to assess options of duration of pregnancy prevention against potential return to personal natural fertility (RTF).

##### 3.6.6.1 Qualitative

Views on acceptable duration of protection and return to natural fertility time were similar across the three countries. Generally, acceptability decreased along with the duration set options; equal length of PP and RTF was also important. However, views differed and there was not one overarching consistent opinion. The main perceived advantage of contraceptive protection was expressed across the range of options: the ability to space births as needed or wanted and to have time to prepare for pregnancy. However, the preferred spacing/time differed between respondents—some wanted as long a time as possible, others wanted a shorter time. The most acceptable option was 6 months' PP with a 12-month RTF time. However, this was not acceptable to around a third of respondents. There were some respondents who felt that the RTF was too short, and for some too long, for most options. Despite the variance in opinions, some disadvantages emerged from shorter durations: affordability concerns, getting pregnant sooner than wanted, and the potential of forgetting to renew the contraceptive. The same duration sets were considered too short or too long, depending on women's current need for spacing. The question on most preferred option was added to the quantitative study after the qualitative responses were analyzed to provide more clarity regarding this complex topic.

##### 3.6.6.2 Quantitative

Respondents in the quantitative study were asked to assess whether each pregnancy prevention and RTF duration set options (Supplementary Figure 6) was acceptable or not; they were then asked to select one of the options they had previously indicated to be acceptable as their preferred option. Respondents were also able to indicate that they had no preferred option. Table 13 shows that the three most acceptable options were 2 (6 + 6), 4 (3 + 3) and 1 (6 + 12). Overall, option 2 (6 + 6) was the option most rated as acceptable, with 57.5% of respondents indicating this (55.9% in Ghana, 61.7% in Kenya, 55.0% in Uganda). Option 4 (3 + 3) was indicated as acceptable by 46.4% of respondents [41.3% in Ghana, 54.1% in Kenya (significantly high), 44.0% in Uganda]. Option 1 (6 + 12) was rated as acceptable by 42.5% of respondents (41.0% in Ghana, 46.2% in Kenya, 40.5% in Uganda).

**Table 13:** Acceptability and preference of pregnancy prevention (PP) and potential return to personal natural level of fertility (RTF) duration sets, %.

	Total	Ghana	Kenya	Uganda
Total sample (*n*=)	927	315	303	309
6 months PP + 6 months RTF (6 + 6)				
Acceptable	57.5	55.9	61.7	55.0
Unacceptable	42.5	44.1	38.3	45.0
Preference	20.2	21.9	18.2	20.4
3 months PP + 3 months RTF (3 + 3)				
Acceptable	46.4	41.3	54.1^b^	44.0
Unacceptable	53.6	58.7	45.9^a^	56.0
Preference	4.7	6.0	3.0	5.2
6 months PP + 12 months RTF (6 + 12)				
Acceptable	42.5	41.0	46.2	40.5
Unacceptable	57.5	59.0	53.8	59.5
Preference	29.2	32.1	33.0	22.7^a^
1 month PP + 1 month RTF (1 + 1)				
Acceptable	41.3	32.4	40.9	50.8^b^
Unacceptable	58.7	67.6^b^	59.1	49.2
Preference	6.7	2.5^a^	6.3	11.3^b^
3 months PP + 6 months RTF (3 + 6)				
Acceptable	34.4	28.6	37.3	37.5
Unacceptable	65.6	71.4^b^	62.7	62.5
Preference	6.4	7.0	5.6	6.5
1 month PP + 3 months RTF (1 + 3)				
Acceptable	31.8	24.1^a^	31.7	39.8^b^
Unacceptable	68.2	75.9^b^	68.3	60.2^a^
Preference	1.0	2.2	1.0	2.6
No preference	28.0	27.9	28.1	28.2

^a^ Significantly lower than the 2 other countries.

^b^ Significantly higher than the 2 other countries.

Respondents were then asked to select their most preferred duration set or select no preference (those who selected no option was acceptable are also included in Table 13 for preference selection data). Option 1 (6 + 12) was most selected, with 29.2% of the overall sample choosing this option [32.1% in Ghana, 33.0% in Kenya, 22.7% in Uganda (significantly low)]. The next most selected options were no preference, at 28.0% overall (27.9 in Ghana, 28.1 in Kenya and 28.2 in Uganda). Option 2 (6 + 6) was preferred by 20.2% overall (21.9% in Ghana, 18.2% in Kenya, 20.4% in Uganda).

### 3.7 Secondary objective

#### 3.7.1 Value proposition

The qualitative component of this study revealed a consistently held set of value propositions. There were three central value propositions for a MAP: ease of use, convenience and discretion. Qualitative discussions illustrated that they were not mutually exclusive and interact with one another. For example, respondents explained that some factors such as self-administration double up as ease of use and convenience value propositions. Furthermore, respondents also stated that something that can be self-administered also means less reliance on the healthcare system and having to spend time accessing HCPs, which was described as the convenience value proposition. Respondents described self-administration as also feeding into a very important value proposition—discretion.

#### 3.7.2 Perceived ease of administration

##### 3.7.2.1 Quantitative

Respondents in the quantitative phase were asked: “Based on the description and picture of the microarray patch/MAP, how easy do you think it will be to administer yourself?” Answer options were given as a 1–5 scale, with 1 being *Very difficult*, 2 being *Somewhat difficult*, 3 being *Neither easy nor difficult,* 4 being *Somewhat easy* and 5 being *Very easy*. 84.6% of the overall sample selected T2B, whereas 6.1% selected B2B. The mean score overall was high, at 4.36 out of 5.0.

Those who thought that it would be difficult to administer a MAP (*n* = 57 or 6.1% out of 927) were asked why. Reasons (coded from open-ended responses) were: that they had never used it before or that it was new (*n* = 15 or 26.3%), that they were not confident in making sure they had administered it correctly (*n* = 13 or 22.8%), that some believed that they needed training first or that it would be better to have an HCP administer the MAP (*n* = 12 or 21.1%) and that the MAP may be painful or too painful (*n* = 9 or 15.8%).

#### 3.7.3 Confidence in MAP

##### 3.7.3.1 Qualitative

Nearly all respondents indicated that they would have confidence in this type of contraceptive. Reasons for this confidence across all three countries were: perceived ease of use and easy application, including self-application, and discretion of the method. In Ghana and Kenya, safety, manageable side effects and no HCP involvement were also given as reasons.

##### 3.7.3.2 Quantitative

The top three reasons are presented here; Supplementary Table S1 shows all reasons. Almost three quarters, 72.9%, of respondents answered “Yes” [77.8% in Ghana, 74.9% in Kenya, 66.0% in Uganda (significantly low)]. “Not sure” was selected by 20.5% of respondents overall, driven by Uganda which was significantly higher than both other countries at 25.6% (with 17.8% in Ghana and 18.2 in Kenya).

Reasons given in an open-ended question coded by the interviewers (with a pre-defined list and “Other—specify”) for having confidence (those selecting option “Yes” which represents *n* = 676 or 72.9% out of 927 total sample) were: ease of use and ease of application (specified by 79.1% of respondents overall), followed by the ability to self-apply (56.1% overall). The third most common answer was duration, which was described as six months’ pregnancy prevention in the CTPP (47.5% overall).

In terms of the top three reasons as to why respondents would not have confidence in this type of contraceptive (Supplementary Table S1, those selecting “Not sure” and “No” which represents *n* = 251 or 27.1% out of the 927 total sample), the most common reason was side effects (56.2% of overall respondents). Effectiveness was selected by 34.7% overall. Safety was the third most common reason, selected by 29.9% overall.

#### 3.7.4 Consistency of use

Respondents were asked whether they would use the MAP consistently. The same wording was used in the quantitative and qualitative phases: “Would you use this contraceptive consistently (all the time), until you wish to conceive/get pregnant? This means that you would reapply a new MAP at the end of each 6-month duration.”

##### 3.7.4.1 Qualitative

For respondents, the MAP must fulfill certain expectations to be used consistently, these being: side effects that are manageable, effectiveness, and regular periods with no excessive bleeding. The majority would use the MAP consistently until they wished to get pregnant; this was on the grounds that it fulfilled its value proposition of being easy to use/apply, discreet, safe to use consistently, and that it would save money.

##### 3.7.4.2 Quantitative

Overall, 81.2% of respondents indicated that they would use the MAP consistently [81.9% in Ghana, 86.5% in Kenya, 75.4% in Uganda (significantly low)]. Whereas 13.5% of the overall sample were not sure whether they would consistently use the MAP [12.1% in Ghana, 9.9% in Kenya, 18.4% in Uganda (significantly high)], and 5.3% of respondents overall indicated that they would not use the MAP consistently (6.0% in Ghana, 3.6% in Kenya, 6.1% in Uganda).

Respondents who indicated that they would not consistently use the MAP were asked to give reasons for which interviewers coded from a pre-defined list with “Other—specify” option. Please note that this is a small sample size (*n* = 49 in total; *n* = 19 in Ghana, *n* = 11 in Kenya, *n* = 19 in Uganda), and no analysis of statistical significance is possible. The most common reason was if side effects were experienced (*n* = 27 overall; *n* = 7 in Ghana, *n* = 7 in Kenya, *n* = 13 in Uganda). The second most common reason was if the respondent's partner was opposed to the MAP (*n* = 10 overall; *n* = 5 in Ghana, *n* = 1 in Kenya, *n* = 4 in Uganda). A total of nine respondents overall (*n* = 2 in Ghana, *n* = 3 in Kenya, *n* = 4 in Uganda) indicated that they would take a break even if they did not experience side effects.

#### 3.7.5 Partner influence

##### 3.7.5.1 Quantitative

Respondents were asked: “How much impact, if any, would your partner's opinion about the microarray patch/MAP have on your desire to use it?”

Four predefined options were given. The most-chosen option, *I would not use the microarray patch/MAP unless my partner was fully supportive*, was selected by 30.9% of respondents overall (33.3% in Ghana, 26.1% in Kenya, 33.0% in Uganda). The second, *I would use the microarray patch/MAP without my partner's knowledge if he wasn't fully supportive*, was selected by 26.9% of respondents overall [32.7% in Ghana (significantly high), 22.8% in Kenya, 24.9% in Uganda]. The third, *I would use the microarray patch/MAP with my partner's knowledge even if he wasn't fully supportive*, was selected by 24.5% of respondents overall [20.0% in Ghana, 31.4% in Kenya (significantly high), 22.3% in Uganda]. The least-chosen option, *I would be hesitant to use the microarray patch/MAP if my partner wasn't fully supportive*, was selected by 17.8% of respondents overall (14.0% in Ghana, 19.8% in Kenya, 19.7% in Uganda). The results are in Supplementary Table S2.

Overall, 51.4% of respondents would use the MAP if their partner was not fully supportive (24.5% with his knowledge and 26.9% without), and 48.7% would either not use it or would be hesitant to use it if their partner was not fully supportive. Slightly more respondents state they are more likely to use without his knowledge than with if not supportive; this could relate to the importance of discretion as part of the perceived value of the MAP.

#### 3.7.6 Access and training on use

Respondents in both phases of the study were asked about where they would like to access the MAP, where they would like to use it, and how they would want to receive training or information on application.

##### 3.7.6.1 Qualitative

Respondents were asked where they would like to obtain the MAP (between healthcare facilities—hospitals or clinics—and pharmacies/drugstores) and where they would use it, including if this would differ between the first application and subsequent uses. In Ghana, around three quarters of respondents preferred to obtain the MAP from pharmacies/drugstores and one quarter from healthcare facilities; in Kenya, the split was roughly even, and in Uganda, the majority preferred healthcare facilities. Reasons for preferring one type of access point were very similar across the three countries. Healthcare facilities were perceived as having knowledgeable staff, being trustworthy, being sources of information, and from a small minority of respondents, that they could trust that the medication supplied there is authentic. Pharmacies/drugstores were perceived as being convenient, familiar, simple, without queues, and discreet; a small minority of respondents in Ghana mentioned being questioned in hospitals but not in pharmacies.

In terms of where to use the MAP, it was almost unanimous in Kenya and Uganda that respondents preferred to use the MAP at the place obtained for the first use, and at home for subsequent applications. The reasons for this were that they could learn how to use it from HCPs and ask questions the first time, and then have the benefit of privacy at home from then on. In Ghana, however, around half of respondents indicated that they would want to use the MAP at home every time, including the first application. The reasons given for this were privacy, and a perception that the MAP would be straightforward to apply provided that it came with instructions.

##### 3.7.6.2 Quantitative

Overall, 48.3% of respondents indicated that they would wish to obtain the MAP from a healthcare facility (19.0% in Ghana (significantly low), 71.9% in Kenya (significantly high), 55.0% in Uganda). A pharmacy/drugstore was preferred by 40.8% of respondents (73.7% in Ghana (significantly high), 19.5% in Kenya (significantly low), 28.2% in Uganda). Other options, which were from a Community Health Worker and from a mobile clinic/health provider, were preferred by 5.9% and 2.2% respectively overall. More respondents in Ghana reported that they had recently obtained their contraceptives from a pharmacy compared to Kenya and Uganda, and fewer respondents in Ghana reported having obtained their contraceptives from a healthcare facility than in Kenya and Uganda.

Respondents were also asked: “Assuming you have to apply the microarray patch/MAP yourself, how would you prefer to receive training or information on how to do it?” *Training from a doctor/nurse/Community Health Worker* was preferred by 56.6% of respondents overall (20.0% in Ghana (significantly low); 62.3% in Kenya, 73.6% in Uganda (significantly high)). *Instructions on packaging* was preferred by 31.1% of respondents overall [61.3% in Ghana (significantly high), 28.8% in Kenya, 14.8% in Uganda]. *Training from a pharmacist* was the least preferred option overall, selected by 12.2% of respondents (18.7% in Ghana, 8.9% in Kenya, 11.6% in Uganda).

### 3.8 Tertiary objective

#### 3.8.1 Interest, intent and improvement

It is important to reiterate that this tertiary objective is indicative in nature, it should be seen as an exploration into an initial understanding of what end-users may think or project. The scope of this study did not include segmentation or determination of uptake through a modeled demand forecast.

#### 3.8.2 Interest in finding out more

##### 3.8.2.1 Quantitative

Respondents were asked how interested they were in finding out more about the MAP, on a scale from 1 to 5, with 1 being *Not at all interested*, 2 being *Not very interested*, 3 being *Neither interested nor uninterested*, 4 being *Quite interested* and 5 being *Very interested*. There was high positive interest (T2B) across all countries, with 80.9% overall (85.1% Ghana, 75.6% in Kenya and 81.9% in Uganda). Looking at the top two scores (ratings 4 and 5), just under half (46.2%) of respondents selected *Very interested* and just over a third (34.7%) selected *Quite interested*. Significantly more respondents in Ghana (60.0%) selected *Very interested*, and in both Kenya and Uganda, significantly more respondents than in Ghana selected *Quite interested* (39.6% and 39.8% respectively). The results are in Supplementary Table S3. The mean score was 4.11 out of 5.0 overall [4.29 in Ghana (significantly high), 3.93 in Kenya and 4.09 in Uganda].

#### 3.8.3 Intention to try

Respondents were asked about their likelihood to try the MAP, using a 1–5 scale, with 1 being *Definitely do not want to try*, 2 being *Probably do not want to try*, 3 being *Might or might not want to try*, 4 being *Probably want to try* and 5 being *Definitely want to try*. The question was phrased as follows: “If you learned about the microarray patch/MAP from a physician, nurse or healthcare worker and it was available to you, how interested would you be in trying it now or at some point in the future?” There was a positive score for intention to try, with 68.6% overall scoring within the T2B (74.0% in Ghana, 64.0% in Kenya and 67.6% in Uganda). Looking into the top two scores (ratings 4 and 5), overall, 29.6% of respondents selected 5: *Definitely want to try* [36.8% in Ghana (significantly high), 22.8% in Kenya, 28.8% in Uganda]. *Probably want to try* (rating 4) was selected by 39.1% of respondents overall (37.1% in Ghana, 41.3% in Kenya and 38.8% in Uganda). The results are in Supplementary Tables S4, S5. The mean score was 3.80 out of 5.0 overall (3.95 in Ghana, 3.66 in Kenya and 3.77 in Uganda).

Reasons for interest in trying given in answer to an open-ended question (by those selecting ratings 4–5) were ease of use, effectiveness, duration, and discretion. Reasons given for unlikelihood or uncertainty to try (by those selecting ratings 1–3) were concerns relating to its application, its newness, potential of side effects, satisfaction with what is already being used and a desire for more information.

#### 3.8.4 Comparative value

##### 3.8.4.1 Qualitative

Respondents reported that a MAP could provide benefits compared to existing long-acting contraception in that it could removes the dependency and need to visit a healthcare facility, which would save time and money (on travelling). Lower HCP involvement was perceived to mean greater privacy. A MAP was perceived as less painful and easier to use than other long-acting contraceptive methods as it would not result in permanent scarring like a contraceptive implant or a deep needle penetration like an intra-muscular injection.

##### 3.8.4.2 Quantitative

Respondents were asked: “To what extent do you feel the microarray patch/MAP is a better form of contraception/family planning, compared to other options you are aware of?” The scale used was 1–5, with 1 being *It offers something worse than other options I know about*, 2 being *It offers nothing better than other options I know about*, 3 being *It offers something slightly better than other options I know about*, 4 being *It offers something noticeably better than other options I know about* and 5 being *It offers something significantly better than other options I know about*. Overall 73.0% of respondents gave a generally positive rating (5, 4 and 3) and 47.0% gave a T2B positive rating (5 and 4) [53.0% in Ghana, 25.7% in Kenya (significantly low) and 61.8% in Uganda (significantly high)]. Overall, 25.4% of respondents selected option 5 [32.1% in Ghana, 12.2% in Kenya (significantly low) and 31.4% in Uganda]. Option 4 was selected by 21.7% of respondents overall [21.0% in Ghana, 13.5% in Kenya (Significantly low) and 30.4% in Uganda (significantly high), option 3 was selected by 26.0% (28.9% in Ghana, 26.7 in Kenya and 22.3 in Uganda)]. The results are in Table 9.

The mean score was 3.37 overall [3.61 in Ghana, 2.76 in Kenya (significantly low) and 3.73 in Uganda] out of 5.0.

Those respondents who selected ratings scale scores 3, 4 and 5 were then asked to explain why in an open-ended question. The top reasons for selecting positive comparative scores for a MAP were perceived as easier to apply/use, self-administration, and removal of need for HCP (convenience), a “long” duration of pregnancy prevention (6 months) and that it is seen as discreet/private. The results are available in Supplementary Tables S6, S7.

## 4 Discussion

This paper describes user insights for a contraceptive MAP focusing on preferred product attributes and the value proposition to inform further product development and eventual introduction and promotion of the method. The most widely preferred attribute set for the contraceptive MAP identified by our study is a hand-applied, circular patch measuring approximately two centimeters in diameter that can be self-administered, is applied to the upper arm, and has signals across all stages of application and removal upon administration of drug. The ideal MAP would be applied within a few seconds, provide contraceptive protection for 6 months, and have a return to natural fertility within 6–12 months after the labeled duration of protection. Conducting this study early in the development of the contraceptive MAP was important so developers can align the product designs to end-user preferences and maximize the potential for uptake once available in the market.

There are several published studies that describe user preferences and attitudes for a contraceptive, HIV prevention or MPT MAP. The studies range in methodology and geographic location, but the findings are similar across all with respect to the size, shape, administration location, skin reaction and duration of protection (Supplementary Tables S8, S9) (39–43). Consistently, smaller, round patches applied to the upper arm and in some cases, the thigh, were preferred across all studies that examined those attributes for a contraceptive, HIV prevention, or MPT MAP (39–42). Similar to other studies, our study found that no visible skin irritation or long-lasting markings were most preferred (42, 43). Participants from two different studies expressed their preference for six months duration of pregnancy protection when presented with the option indicating a desire for a longer-acting option (41–43). Furthermore, our study confirms that one patch is preferred over multiple patches to achieve the duration of protection as multiple patches are seen to be less discreet with greater potential for pain or increased side effects (39, 42).

Two other studies found that the MAP can be perceived as “too good to be true” when it is described as painless with little or no skin rash and a short administration or wear time (39, 41). For this reason, developers are exploring ways to assure end-users that the product has been applied correctly including signals like clicking sounds and color change (39, 41, 42). Our study demonstrated a preference for a color change or clicking sound at all three stages of application. Yet, concerns were raised about the clicking sound being too loud to be discreet or for the color change to disappear too quickly leading to user error. It will be important for developers to continue to test the feedback mechanisms for their MAP product with actual use to ensure it meets end-user expectations around discretion and confidence with administration.

Self-administration of the MAP could enhance its appeal to end-users and the health system alike for multiple indications and overall, our results and those of other studies demonstrate a preference for self-administration with some variation (39, 41, 42). Participants' preference for administration, location to obtain, and how to learn more about the MAP aligned within each country and with current contraceptive seeking practices. In Ghana, there was a significant preference for a MAP that is self-administered and obtained from a pharmacy/drugstore with information on self-administration as part of the packaging instructions to maintain privacy. Participants from Uganda preferred administration by an HCP and participants from Kenya equally preferred self-administration and self-administration only after an initial application by an HCP. Both Kenyan and Ugandan participants preferred to obtain the MAP from a healthcare facility with training and information from an HCP as a trusted source. Although most study participants stated they currently receive contraceptives from a healthcare facility, the proportion who receive from a pharmacy was significantly higher in Ghana, which aligns with the preference for pharmacy/drugstore availability of the MAP and reluctance to attend hospitals. These results signal the need for wide availability of the method through both public and private sector outlets including pharmacies/drugstores along with appropriate training, support, and materials to HCPs and women to be able to effectively access and use the method for self-administration.

In Uganda, there was also a significant preference for use of an applicator to administer the MAP although the quantitative results indicated an overall preference for a sticky-back patch only (no applicator). In the MaxDiff exercise, participants from Uganda also expressed a greater tolerance for higher levels of pain and greater skin reaction than in Ghana or Kenya. These differences coupled with the preference for access and administration by an HCP could indicate the need for greater familiarity and assurance of the MAP product and appropriate training and knowledge that the MAP is applied correctly and working as indicated with certain groups.

Our study builds upon existing knowledge of common concerns with hormonal contraception more broadly. Studies consistently show that non-use and discontinuation of modern contraceptives is often associated with health reasons linked to side effects that impact and alter one's natural menstruation (44, 45). The potential menstrual side effects associated with the MAP as described in the CTPP included amenorrhea/no bleeding or irregular bleeding including infrequent, frequent, prolonged or heavy, but would likely resolve to regular periods or amenorrhea over time. In our study, both amenorrhea and irregular bleeding were unacceptable to most with some significant differences between countries. A significantly higher percentage of participants from Kenya perceived amenorrhea to be acceptable while a significantly higher percentage of participants from Ghana perceived it to be unacceptable. Study participants in Uganda found irregular bleeding to be significantly more unacceptable than in Ghana and Kenya. Furthermore, women in our study who reported not having confidence in the contraceptive MAP indicated this was due to concerns around the associated side effects, and for a small percentage of women, they said they would not use the method consistently if side effects were experienced. A study in India and Nigeria also looked at the effect on menstruation of a contraceptive MAP using a discrete choice methodology and the results overwhelmingly showed that a change in menstruation negatively influenced women's interest in the MAP (43).

Another side effect that women and adolescents are concerned about when choosing a contraceptive method is return to fertility and fear of being infertile if there are significant or unknown delays in return to natural fertility (46–48). However, our findings and those from Gualeni et al. did not provide a clear preference for an acceptable duration for return to natural fertility given changes in women's lives and pregnancy intentions (41). It is important to consider the level of confidence in the anticipated duration to return to fertility and counsel women appropriately.

A challenge and opportunity identified in our study and other studies is the variation of preferences across product attributes where up to a third of study participants preferred another attribute or design option. This provides developers with room for variation in the design of the final MAP, which is important given technological feasibility. It also implies that there would be end-user interest in multiple products to provide a range of options including how the MAP is applied (applicator or hand), duration of protection, application or wear time, and duration for return to natural fertility.

### 4.1 Additional research

The contraceptive MAP products are still early in development, and it will be important to re-test their appeal following any product design updates or changes. More qualitative research would help uncover gaps in understanding potential application of the MAP by hand vs. the MAP plus an applicator, applying one or more patches, overall patch size, or desired time to return to fertility. A more detailed exploration of women's perceptions of or interest in the method once the side effect profile is better understood will be critical given the negative reaction to menstrual side effects such as amenorrhea and irregular bleeding. Other areas to test with end-users include instructions for use, particularly for self-administration, and packaging appeal. Messaging to end-users and appropriate counseling messages for HCPs will be important to explore, particularly around addressing concerns related to product safety and side effects.

More research is also warranted to understand the perspective of different stakeholders that influence women's decision making around contraception. HCPs are a key group as they can be guides or gatekeepers for women seeking contraception and as indicated by this research, are often a trusted source of information on new contraceptive methods. Further research in understanding how to support women who are reluctant to enter some healthcare settings is also relevant. Additional research with partners is important to understand their support in or objections to the use of a new method. Partners play a complex role and understanding what they think about the MAP. This will be critical to effectively communicate with them to garner support and reduce the need for concealing contraceptive use as nearly half of study respondents would hesitate to use the MAP if their partner were opposed.

As MAP products develop and the options are narrowed, commercial focus is critical. Data on acceptable pricing for procurers and end-users is important, as well as segmentation and demand forecasting to support decision making on funding and manufacturing needs. Demand forecasts should also take into consideration all current contraceptive options on the market to understand the shifts in the method mix with the introduction of the MAP. Furthermore, research to influence country policies around accessibility and availability of the method, particularly for self-administration, may be necessary.

## 5 Limitations

There are several limitations to this study. First, participants in our study did not use or try the method. Several participants raised concerns on the safety and side effect profile, which could not be tested at the time of this research. There is inherent potential for periods to either stop or become irregular when using the contraceptive MAP. This is due to giving progestin continuously for six months (49). More clinical data to define the product's safety and side effect profile is needed before reassessing end-user preferences.

We do not recommend drawing broad conclusions across and between different country populations. The qualitative portion of the study is indicative in nature, and further, qualitative samples often fluctuate as not all questions are asked or answered. The scope of our research did not include development of data for a segmentation analysis, a forecast or modeling, product pricing or price sensitivity evaluation. The research and the flow of the stimuli was organized in such a way that we cannot concretely answer questions about the preference for two patches, patch size, reasoning for applicator use, or choice on desired time to return to fertility. Reversing the questions on one vs. two patches may influence responses and preference of two patches over one. Showing only larger size patches that are likely more technically feasible may have rendered different responses. Introducing the use of an applicator with the patch sooner may also have resulted in different levels of acceptability for an applicator.

In terms of the body map stimuli the middle/center/front of the thigh was omitted. Respondents were given the options for upper inner or outer thigh; however, the middle/center/front of the thigh is a credible location for MAP application.

Finally, our study only reflects women's perceptions of the MAP product category and does not explore trade-offs with other current or new contraceptive methods. Contraceptive choice is paramount for quality family planning programming and any new method introduced will attract users who have not previously used a method, have discontinued or have switched from an existing method. Understanding end-users' rationale for making trade-offs among many contraceptive products is important for decision-making for family planning programs and funding.

## 6 Conclusion

The results across the three countries demonstrate that the contraceptive MAP has a high and broad level of appeal amongst all groups of women who participated in the study and has a strong value proposition around important contraceptive needs such as ease of use, convenience, and discretion. This study confirms women's preferences from other studies for product-specific attributes of a contraceptive, HIV prevention or MPT MAP such as shape, size, location of administration and duration of protection while also understanding preferences for additional attributes and other aspects that define its value proposition in the contraceptive method mix. The majority of study participants responded positively to the option for self-administration as it encompassed all three aspects of the method's value proposition. Future research should include actual use of the product to understand end-users' perspectives on the side effects, feedback mechanism, and pain or skin reaction to provide more feedback on the acceptability of those product aspects.

## Data Availability

The original contributions presented in the study are included in the article/Supplementary Materials, further inquiries can be directed to the corresponding author.
