# Supplementary material for: End-user research into understanding perceptions of and reactions to a microarray patch (MAP) for contraception among women in Ghana, Kenya and Uganda
Source: Front Reprod Health. 2024 Mar 7;6:1351692. doi: 10.3389/frph.2024.1351692 (PMC10954799; doi:10.3389/frph.2024.1351692)
Supplement: Supplementary file 1 [file Datasheet1.docx]

Supplementary Material

**Social and behavioral end-user research with women in Ghana, Kenya and Uganda understanding perceptions and reactions to a Microarray Patch (MAP) which delivers six months of contraception.**

Moushira El-Sahn*, Rose Elliott, Mona El-Sahn, Jeff Lucas , and Trisha Wood Santos

*** Correspondence:** Corresponding Author: Moushira El-Sahn [Moushira@routes2results.org](mailto:Moushira@routes2results.org)

**ABBREVIATIONS**

CAPI Computer Assisted Personal Interviewing

CTPP Consumer Target Product Profile

CHW Community Health Worker

DMPA-IM Depo-Provera Medroxyprogesterone acetate (DMPA) Intermuscular

FP Family Planning

HCP Healthcare Practitioner

HIV Human Immunodeficiency Virus

IDI In-Depth Interview

IUD Intrauterine Device

MAP Microarray Patch

MPT Multipurpose Prevention Technology

PP Pregnancy Prevention

RTF Return to natural Fertility level

R2R Routes2Results

SEC Socio Economic Class

STI Sexually Transmitted Infection

WRA Women of Reproductive Age

# Supplementary Figures and Tables

**Supplementary Figure 1. Five tailor-designed visual graphic shows and script for introducing the concept of a MAP**


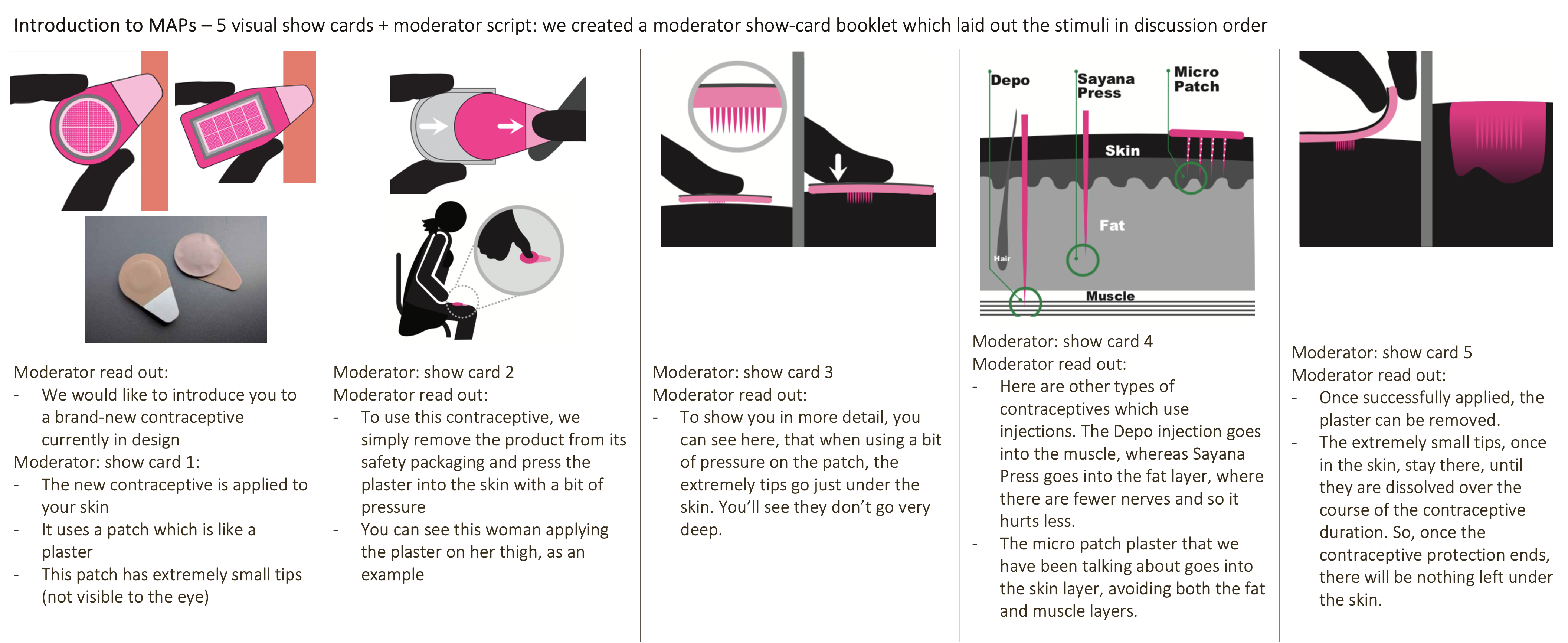


**Supplementary Figure 2. Consumer Target Product Profile (CTPP) stimuli show card**

Information given to every respondent about MAPs


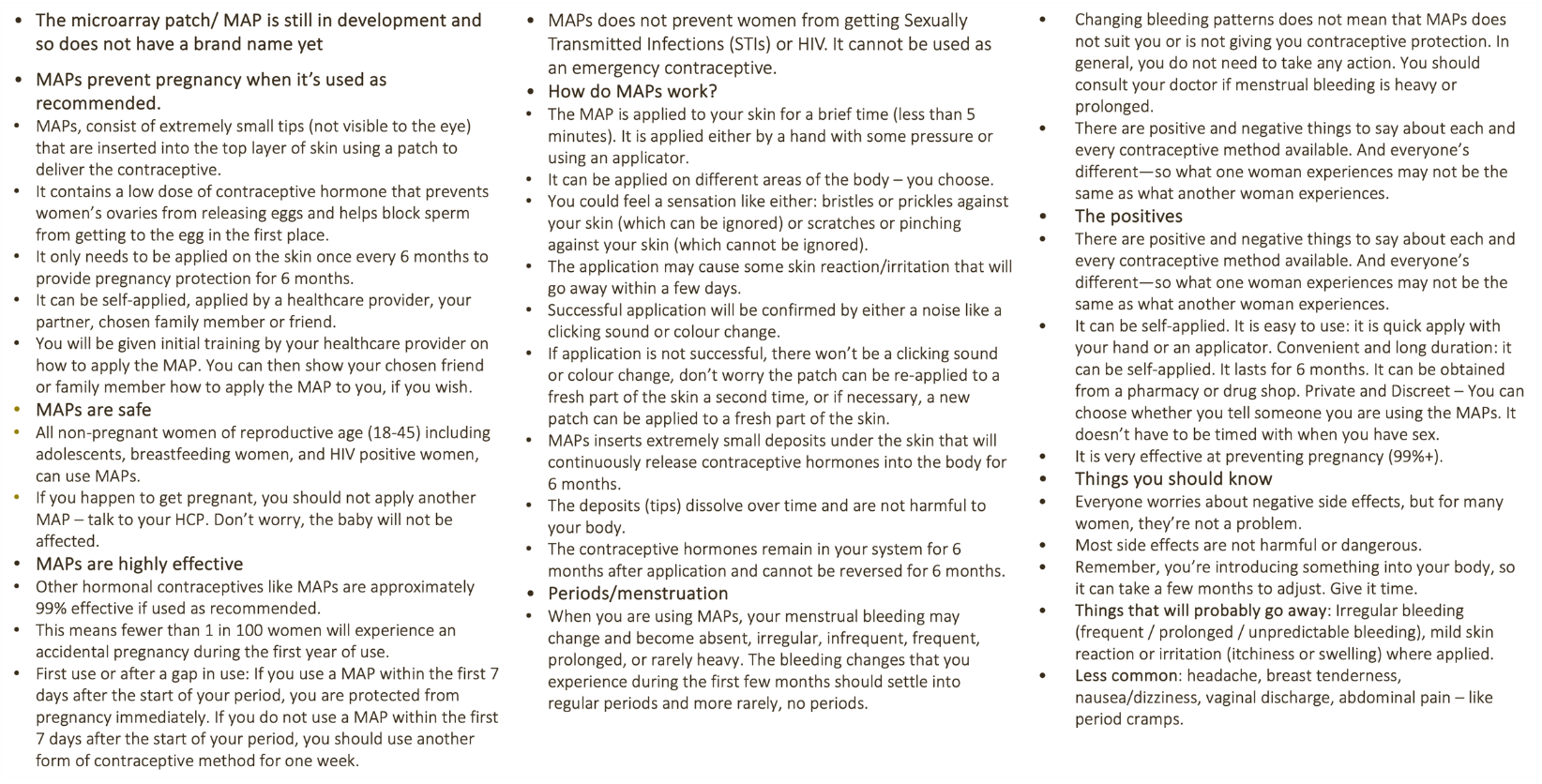


**Supplementary Figure 3. Shape options stimuli**

Circular (left), rectangular (right)


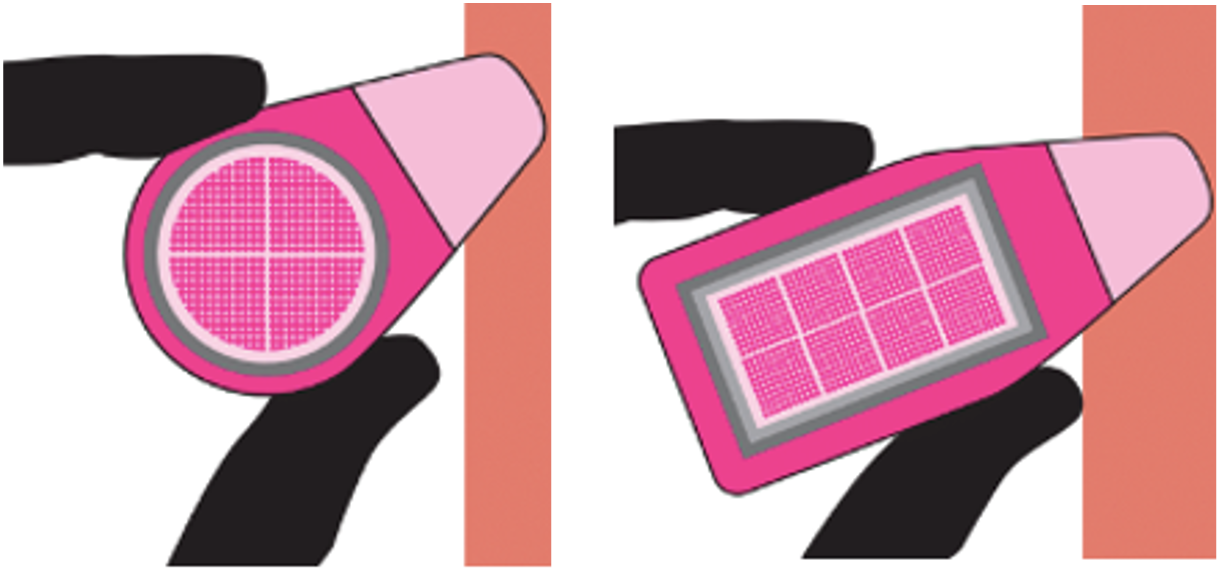


**Supplementary Figure 4. Size options stimuli**

Option 1: 2cmx2cm, option 2: 3cmx3cm, option 3: 5cmx5cm

Shown to scale to respondents


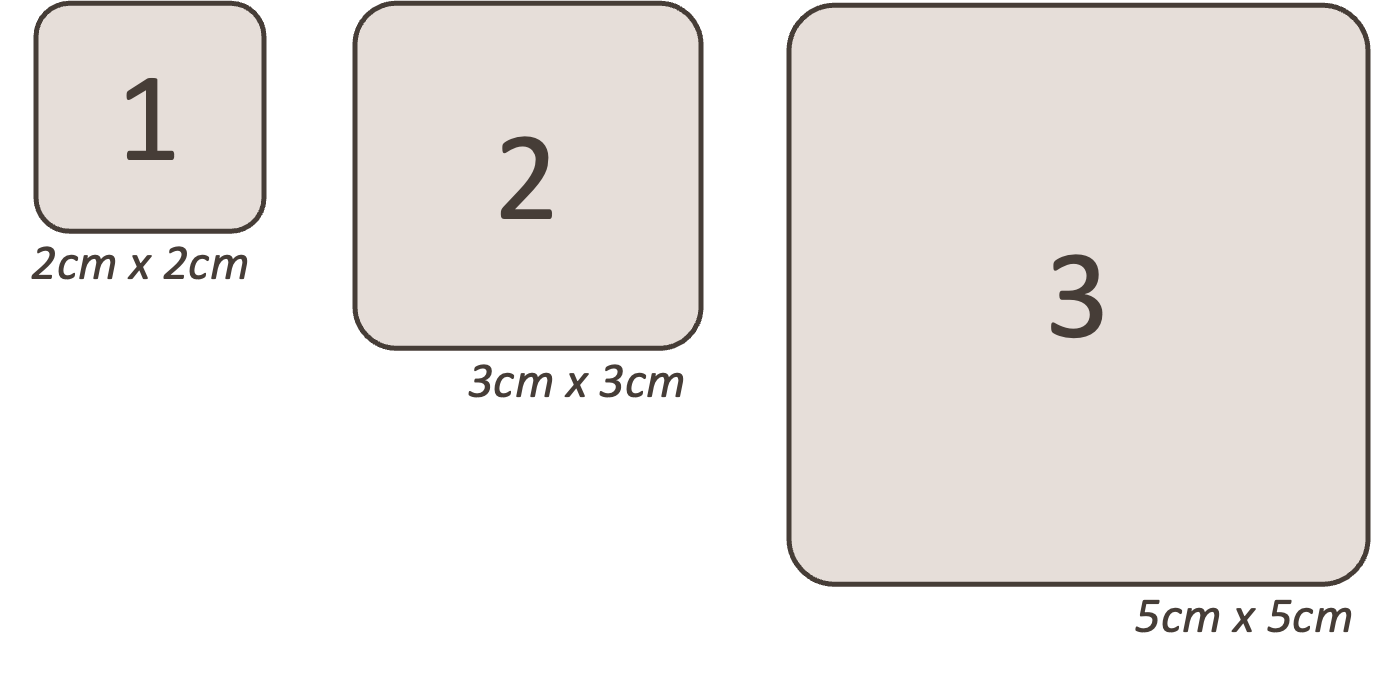


**Supplementary Figure 5. Body MAP stimuli for MAP site of application questions**


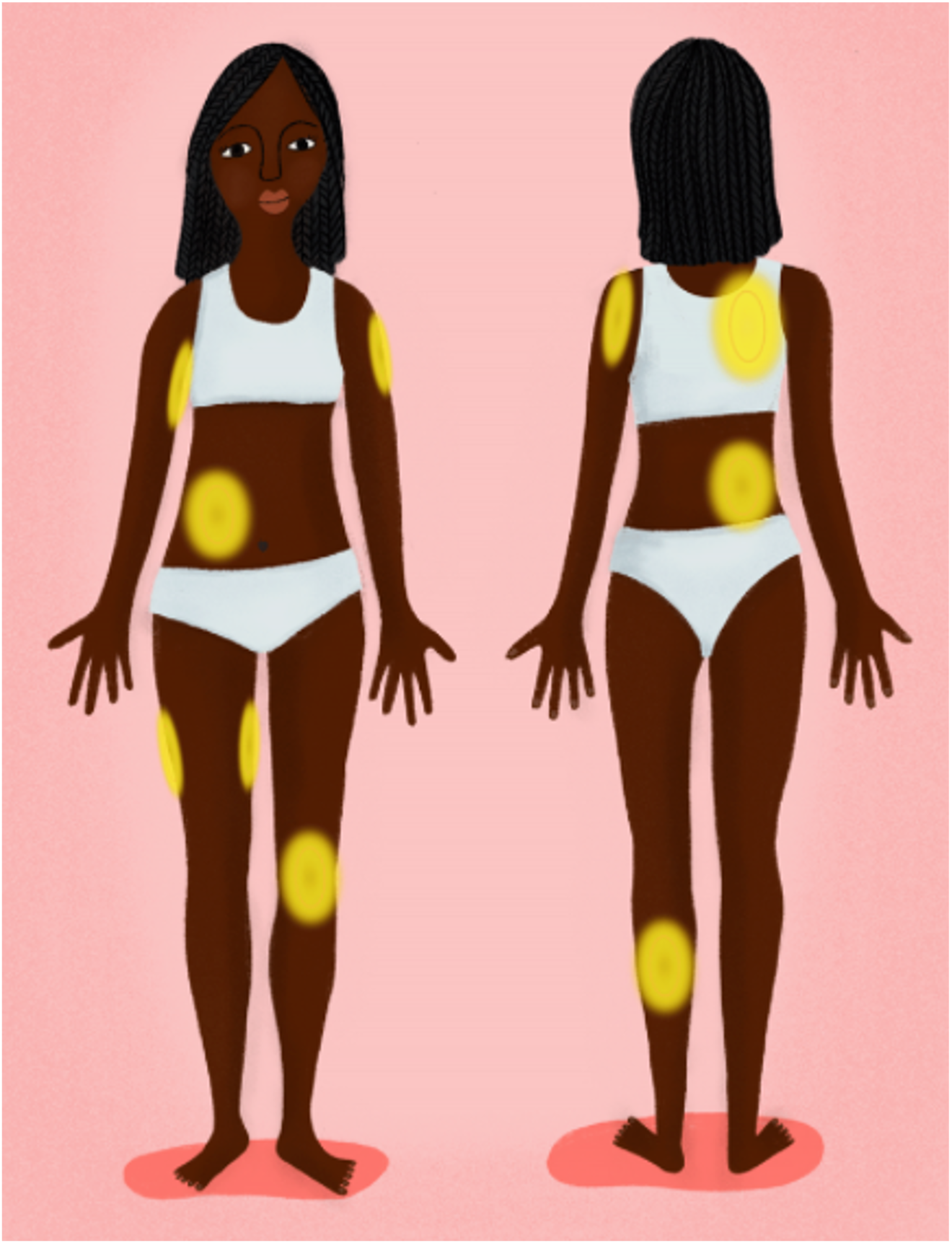


**Supplementary Figure 6. Stimuli show card used to describe the duration sets of pregnancy prevention (PP) and return to personal natural level of fertility**


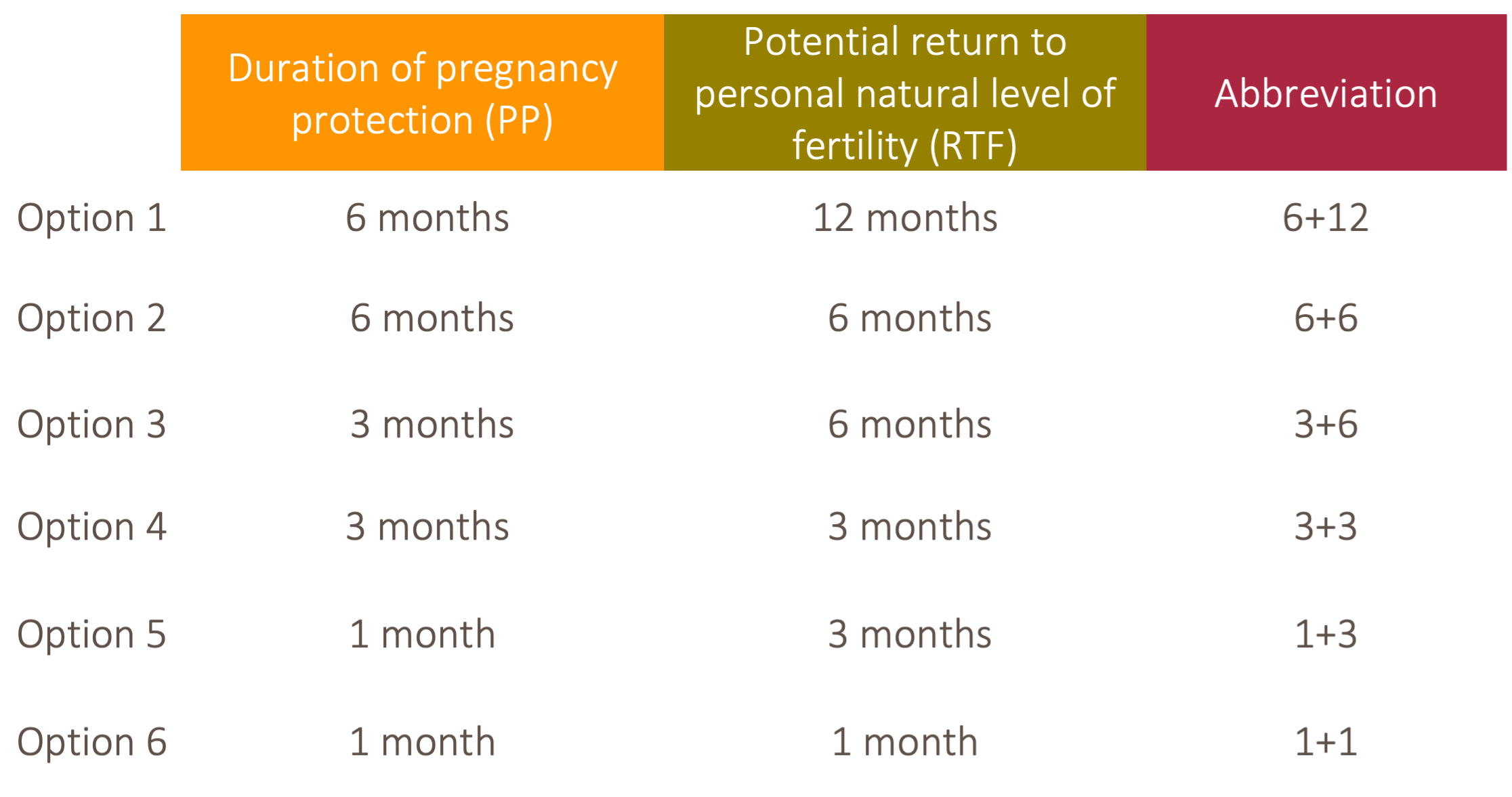


**Supplementary Table 1. Reasons why not confident/ unsure (%)**

|  | **Total** | **Ghana** | **Kenya** | **Uganda** |
| --- | --- | --- | --- | --- |
| **Respondents confident in a MAP (n=)** | 676 | 245 | 227 | 204 |
| Side effects | 56.2 | 58.6 | 46.1 | 61.9 |
| Effectiveness | 34.7 | 44.3 | 31.6 | 30.5 |
| Safety | 29.9 | 38.6 | 18.4↡ | 32.4 |
| Duration | 21.9 | 35.7↟ | 10.5↡ | 21.0 |
| Concerns of ease of use | 20.3 | 30.0 | 11.8 | 20.0 |
| Self-application | 19.1 | 17.1 | 15.8 | 22.9 |
| Accessibility | 14.7 | 17.1 | 11.8 | 15.2 |
| Discreetness | 12.4 | 18.6 | 10.5 | 9.5 |
| New/untested | 8.8 | 2.9 | 19.7↟ | 4.8 |
| Other | 5.2 | 1.4 | 5.3 | 7.6 |
| ↡significantly lower than the 2 other countries  ↟significantly higher than the 2 other countries | | | | |

**Supplementary Table 2: Partner influence statement agreement (%)**

|  | **Total** | **Ghana** | **Kenya** | **Uganda** |
| --- | --- | --- | --- | --- |
| **Total sample (n=)** | 927 | 315 | 303 | 309 |
| I would not use the MAP unless my partner was fully supportive | **30.9** | **33.3** | 26.1 | **33.0** |
| I would use the MAP without my partner’s knowledge, if he wasn’t fully supportive | 26.9 | **32.7↟** | 22.8 | 24.9 |
| I would use the MAP with my partner’s knowledge, even if he wasn't fully supportive | 24.5 | 20.0 | **31.4↟** | 22.3 |
| I would be hesitant to use the MAP if my partner wasn't fully supportive | 17.8 | 14.0 | 19.8 | 19.7 |
| ↡significantly lower than the 2 other countries  ↟significantly higher than the 2 other countries | | | | |

**Supplementary Table 3. Interest in finding out more (%)**

|  | **Total** | **Ghana** | **Kenya** | **Uganda** |
| --- | --- | --- | --- | --- |
| **Total sample (n=)** | 927 | 315 | 303 | 309 |
| Top 2 positive interest rating score (5+4) | 80.9 | 85.1 | 75.6 | 81.9 |
| **5 = very interested** | **46.2** | **60.0↟** | **36.0** | **42.1** |
| 4 = quite interested | 34.7 | 25.1↡ | **39.6** | 39.8 |
| 3 = neither interested nor uninterested | 8.2 | 5.1 | 12.2↟ | 7.4 |
| 2 = not very interested | 5.4 | 3.5 | 5.9 | 6.8 |
| 1 = not at all interested | 5.5 | 6.3 | 6.3 | 3.9 |
| Bottom 2 negative interest rating score (1+2) | 10.9 | 9.8 | 12.2 | 10.7 |
| Mean | 4.11 | 4.29↟ | 3.93 | 4.09 |
| ↡significantly lower than the 2 other countries  ↟significantly higher than the 2 other countries | | | | |

**Supplementary Table 4. Intent on trying a MAP if told by an HCP it was available (%)**

|  | **Total** | **Ghana** | **Kenya** | **Uganda** |
| --- | --- | --- | --- | --- |
| **Total sample (n=)** | 927 | 315 | 303 | 309 |
| Top 2 positive intent rating score (5+4) | 68.6 | 74.0 | 64.0 | 67.6 |
| 5 = definitely want to try | 29.6 | 36.8 ↟ | 22.8 | 28.8 |
| 4 = probably want to try | 39.1 | 37.1 | 41.3 | 38.8 |
| 3 = might or might not want to try | 20.0 | 16.5 | 23.1 | 20.4 |
| 2 = probably do not want to try | 4.2 | 2.9 | 5.3 | 4.5 |
| 1 = definitely do not want to try | 7.2 | 6.7 | 7.6 | 7.4 |
| Bottom 2 negative interest rating score (1+2) | 11.4 | 9.5 | 12.9 | 12.0 |
| Mean | 3.80 | 3.95 | 3.66 | 3.77 |
| ↡significantly lower than the 2 other countries  ↟significantly higher than the 2 other countries | | | | |

**Supplementary Table 5. Reasons for intent trying / not trying MAP (%)**

|  | **Total** | **Ghana** | **Kenya** | **Uganda** |
| --- | --- | --- | --- | --- |
| **Positive intent on trying (4+5) MAP (n=)** | 636 | 233 | 194 | 209 |
| Easy/simple to use | 20.8 | 22.7 | 22.2 | 17.2 |
| Effectiveness | 14.9 | 25.3↟ | 8.8 | 9.1 |
| Duration/it lasts 6 months | 11.8 | 6.4 | 18.6↟ | 11.5 |
| Offers privacy/discreet | 9.6 | 7.3 | 17.5↟ | 4.8 |
| **Negative intent on trying (1-3) MAP (n=)** | 291 | 82 | 109 | 100 |
| It’s application | 11.0 | 12.2 | 13.8 | 7.0 |
| It’s new | 9.3 | 8.5 | 1.8↡ | 9.0 |
| It might have side effects | 6.2 | 8.5 | 1.8↡ | 9.0 |
| Others need to use it first / recommend it first | 6.2 | 6.1 | 6.4 | 6.0 |
| I prefer/satisfied with what I am using now | 5.8 | 2.4 | 3.7 | 11.0↟ |
| I want to know more about how it works | 5.8 | 1.2↟ | 7.3 | 8.0 |
| ↡significantly lower than the 2 other countries  ↟significantly higher than the 2 other countries  Only mentions over ~10% overall included | | | | |

**Supplementary Table 6. Whether a MAP is a better form of contraception compared to other options aware of (%)**

|  | **Total** | **Ghana** | **Kenya** | **Uganda** |
| --- | --- | --- | --- | --- |
| **Total sample (n=)** | 927 | 315 | 303 | 309 |
| Top 3 positive rating score (5,4+3) | 73.0 | 81.9 | 52.5↡ | 84.1 |
| Top 2 positive rating score (5+4) | 47.0 | 53.0 | 25.7↡ | 61.8↟ |
| 5 = offers something significantly better | 25.4 | 32.1 | 12.2↡ | 31.4 |
| 4 = offers something noticeably better | 21.7 | 21.0 | 13.5↡ | 30.4↟ |
| 3 = offers something slightly better | 26.0 | 21.0 | 26.7 | 22.3 |
| 2 = offers nothing better | 18.4 | 11.1 | 32.7↟ | 15.9 |
| 1 = offers something worse | 8..3 | 6.7 | 14.5↟ | 3.9 |
| Bottom 2 negative rating score (1+2) |  |  |  |  |
| Mean | 3.37 | 3.61 | 2.76↟ | 3.73 |
| ↡significantly lower than the 2 other countries  ↟significantly higher than the 2 other countries | | | | |

**Supplementary Table 7. Top reasons why MAP is better form of contraception (%)**

|  | **Total** | **Ghana** | **Kenya** | **Uganda** |
| --- | --- | --- | --- | --- |
| **Respondents think MAP is better (1-3) MAP (n=)** | 802 | 285 | 256 | 261 |
| Easier to apply/use | 27.5 | 29.5 | 26.1 | 26.9 |
| Self-administration / do not need HCP | 25.6 | 30.5 | 29.0 | 17.24↡ |
| Last long/ 6 months duration | 11.7 | 4.1↡ | 18.8↟ | 12.3 |
| It’s discreet/private | 10.6 | 11.1 | 15.5% | 5.2↡ |
| ↡significantly lower than the 2 other countries  ↟significantly higher than the 2 other countries  Only mentions over ~5% overall included, very little collective consistency in responses | | | | |

**Supplementary Table 8. User Preference Studies for Contraceptive MAP**

Study population, methodologies and product indication

| Author, year, study countries | Study population | Methodology used | Product indication |
| --- | --- | --- | --- |
| Ismail et al. 2023 (14)  South Africa, Uganda | Adolescent girls and young women (AGYW), female sex workers (FSW), men who have sex with FSW, men who have sex with men (MSM), HCPs, policymakers and SRH experts | Focus Group Discussions  Mock-use Exercises with In-depth Interviews  Key Informant Interviews | MAP for HIV prevention alone and in combination with contraceptive |
| Gachigua et al. 2023 (15)  Kenya | AGYW, FSW, MSM, male partners of AGYW and FSW | Focus Group Discussions  Mock-use Exercises with In-depth Interviews  Self-administered Questionnaire  Key Informant Interviews | MAP for HIV prevention alone and in combination with contraceptive |
| Gualeni et al. 2022 (19)  Uganda  Gambia  UK  Malawi | Women and trans-males of reproductive age, their partners, HCPs and SRH Experts | Focus Group Discussions  Semi-structured Interviews | Contraceptive MAP |
| Brunie et al. 2019 (17)  India  Nigeria | Women of reproductive age and family planning providers | Focus Group Discussions  In-depth Interviews | Contraceptive MAP |
| Callahan et al. 2021 (18)  India  Nigeria | Women of reproductive age | Discrete Choice Experiment | Contraceptive MAP |
| Li et al. 2019 (8)  USA | Women of reproductive age | Tolerability and acceptability of use of MAP | Placebo MAP |

**Supplementary Table 9. Comparison of Preferred MAP Attributes.** Attribute options and levels were different across the studies and the results cannot be compared quantitatively.

| MAP Attribute | Our Study (2024)  Ghana, Kenya, Uganda | Ismail et al. (2023) (14)  South Africa, Uganda | Gachigua et al. (2023) (15)  Kenya | Gualeni et al. (2022) (19)  Uganda, Gambia, UK, Malawi | Callahan et al. (2021) (18)  India, Nigeria | Brunie et al. (2019) (17)  India, Nigeria |
| --- | --- | --- | --- | --- | --- | --- |
| Shape and Size | Circular with 2cm diameter | Smallest prototype or smaller | Smaller than prototype |  | Smallest choice | Circular with 2.5cm-3.8cm (1in-1.5in) diameter |
| Site of Application | Upper arm | Upper arm or thigh | Forearm, Upper arm or thigh | Upper arm and thigh | Foot | Upper arm or thigh (non-visible) |
| Duration of Protection | 6 months | 1-month minimum duration (some preferred 2-3 months or longer) | Various:  1 month (AGYW)  3-12 months (FSWs) | 6 months | 6 months | 3-6 months |
| Number of Patches | 1 patch | 1 patch |  |  |  | 1 patch |
| Feedback Signal | Color change or click | Wanted improvement with color change or other visual cue |  | Visual and audio confirmation |  | Click sound |
| Application Sensation | Bristles/prickles against skin |  |  |  | No pain | Not more painful than injection and no residual pain |
| Skin Reaction | No visible skin irritation and small welts disappear within 1 day |  |  |  | Rash lasting ≤ 1 day | None or a rash or mark lasting ≤ a few hours |
| Application/Wear Time | A few seconds | 30 minutes with option for longer with greater duration of protection | 1-10 minutes |  |  | 1-5 minutes |
| Administration/  Administrator | Mixed for self-administration and learning from HCP before self-administration | Learn from HCP first and then self-administration |  | HCP in UK, Malawi, Gambia  Learn from HCP first and then self-administration in Uganda |  | Mixed for self-administration and learning from HCP before self-administration |
| Effect on Menstruation | Maintain regular menstruation over irregular bleeding or amenorrhea although some would tolerate some irregular bleeding |  |  |  | Maintain regular menstruation over irregular bleeding or amenorrhea |  |
| Return to Fertility | 6-12 months most preferred |  |  | No consensus, large proportion preferred 0-3 months |  |  |
